# Polymorphic self-assembly of helical tubules is kinetically controlled†

**DOI:** 10.1039/d2sm00679k

**Published:** 2022-08-12

**Authors:** Huang Fang, Botond Tyukodi, W. Benjamin Rogers, Michael F. Hagan

**Affiliations:** Martin Fisher School of Physics, Brandeis University, Waltham Massachusetts 02454 USA hagan@brandeis.edu; Department of Physics, Babes-Bolyai University 400084 Cluj-Napoca Romania

## Abstract

In contrast to most self-assembling synthetic materials, which undergo unbounded growth, many biological self-assembly processes are self-limited. That is, the assembled structures have one or more finite dimensions that are much larger than the size scale of the individual monomers. In many such cases, the finite dimension is selected by a preferred curvature of the monomers, which leads to self-closure of the assembly. In this article, we study an example class of self-closing assemblies: cylindrical tubules that assemble from triangular monomers. By combining kinetic Monte Carlo simulations, free energy calculations, and simple theoretical models, we show that a range of programmable size scales can be targeted by controlling the intricate balance between the preferred curvature of the monomers and their interaction strengths. However, their assembly is kinetically controlled—the tubule morphology is essentially fixed shortly after closure, resulting in a distribution of tubule widths that is significantly broader than the equilibrium distribution. We develop a simple kinetic model based on this observation and the underlying free-energy landscape of assembling tubules that quantitatively describes the distributions. Our results are consistent with recent experimental observations of tubule assembly from triangular DNA origami monomers. The modeling framework elucidates design principles for assembling self-limited structures from synthetic components, such as artificial microtubules that have a desired width and chirality.

## Abstract 2

Dynamical simulations, free energy calculations, and analytical theory of helical tubule self-assembly show that the morphology distribution of assembled tubules is kinetically controlled.

## Graphical abstract

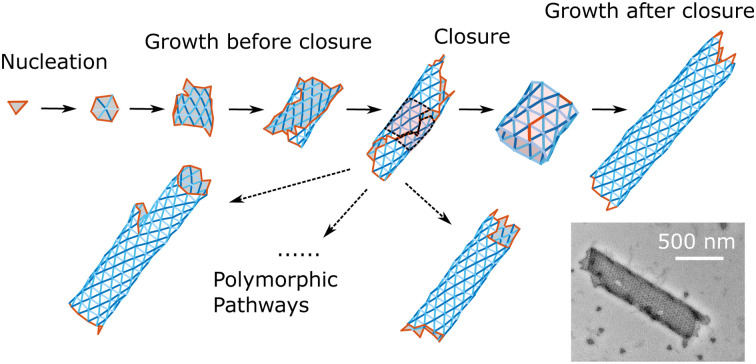

## I. Introduction

Many biological functions rely upon the assembly of self-limited structures that have well-defined finite sizes, and yet are much larger than the size of the individual building blocks. Examples include the assembly of protein capsomers into viral shells with the appropriate size to encapsulate the viral nucleic acid, assembly of tubulin into microtubules with diameters that confer sufficient rigidity to mechanically support the cell,^1,2^ and, within butterfly wings, the organization of chitin into nanostructured domains on the scale of visible light to make the tissue iridescent.^3,4^ In contrast, most structures assembled from synthetic building blocks undergo *unlimited* growth into crystals or amorphous materials.^5–7^ The biological structures described above are examples of ‘curvature-controlled’ assemblies, in which the building blocks assemble with a preferred curvature that leads the structure to close upon itself in one or more directions.

There has been an intense interest in mimicking such functional biological structures by developing synthetic building blocks that can be pre-programmed to assemble with curvatures leading to self-closure. To this end, researchers have recently used DNA origami (*e.g.*^8,9^) and protein design (*e.g.*^10–12^) to engineer building blocks that assemble into polyhedral capsids or tubules with designed diameters. However, due to thermal fluctuations and kinetic effects, assembled structures typically exhibit polymorphism in the limited dimension rather than a single well-defined diameter.^13–15^ Understanding the factors that control this size distribution is essential for achieving functional self-limited assemblies. In this article, we use computer simulations and kinetic models to understand the dynamical pathways of helical tubule assembly, and the resulting polymorphic distribution of assembled tubule structures.

Curvature-controlled assemblies in biology frequently rely on symmetry principles to maximize their ‘economy’ of assembly, meaning the size of the structure that can be assembled for a given number of distinct subunit species.^15^ For example, icosahedral symmetry maximizes the number of identical subunits (60) that can be used to assemble a shell, and many viruses assemble icosahedral capsids.^16–19^ In this sense, helical tubules are even simpler than icosahedral capsids—there is an infinite family of helical tubules with different diameters and pitches, each of which can be assembled from a single subunit species with identical conformations throughout the structure. However, because the subunit curvature changes only slightly between different tubule structures with similar geometries within this family, tubule assembly is highly susceptible to polymorphism. That is, when subunits associate with imperfect geometries during assembly, and these imperfections fail to anneal before becoming trapped by further subunit association, the resulting assembled structures deviate from the ground state tubule structure. Consequently, the geometry distribution of tubules assembled in a finite time depends on a competition between kinetic and thermodynamic factors, and can differ significantly from the equilibrium distribution. Identifying these factors from experiments alone is challenging because most intermediate structures are transient and present at concentrations which are too low to experimentally detect or characterize.

Computer simulations can help to understand self-limited size distributions by revealing the dynamical pathways leading to assembly. However, in comparison to the extensive body of theoretical and computational modeling of icosahedral capsids or shells (*e.g.*^20^), there has been relatively limited study of tubule assembly (*e.g.*^21–29^). Thus, the mechanisms controlling tubule assembly and closure have yet to be completely explored.

In this article, we perform kinetic Monte Carlo simulations on a model of triangular subunits motivated by recent experiments demonstrating the assembly of DNA origami building blocks into helical tubules.^9^ By comparing the distribution of dynamically assembled tubules with equilibrium results, we find that the size and morphology distribution is kinetically controlled. In particular, the structural ensemble is typically quenched shortly after a nascent assemblage first closes upon itself to form a cylindrical tubule. Through a combination of dynamical simulations, free-energy calculations, and simple analytical models, we determine how the resulting size distribution depends on control parameters such as the bending modulus and the pre-programmed target curvature. These results may guide the experimental design of more efficient and accurate self-assembling artificial tubule structures.

The remainder of the article is organized as follows: In Section II A and B, we introduce the kinetic Monte Carlo algorithm that we use to model tubule self-assembly. We then discuss the predicted assembly trajectories and geometry distribution of assembled tubules. In Section II C, we compare simulation outcomes to observations from experiments on tubules self-assembled from DNA origami subunits, and obtain an estimate of the bending rigidity in the experimental system. In Section III A–C, we present calculations of the equilibrium tubule geometry distribution and, through comparison with simulation results, show that the assembled geometry distribution is kinetically controlled. In Section III D–E, we construct a kinetic model that captures these dynamical effects, and use it to predict the assembly behavior as a function of the control parameters. Finally, in Section IV, we discuss implications for future experiments, as well as limitations and possible extensions of the model.

## II. Simulations

### A. Computational model

In our model, monomers are triangular structures composed of three vertices connected by harmonic bonds. We choose a triangle geometry because it closely matches the DNA origami subunits in the motivating experiments,^8,9^ and similar models based on triangular monomers have been recently developed for elastic membranes,^30^ icosahedral shell self-assembly,^31–35^ and geometrically frustrated finite-length tubule-like structures.^36^ The model and simulation algorithm can be readily extended to other monomer geometries; for example, Mohajerani *et al.*^37^ modeled the assembly of hepatitis B virus (HBV) capsids from protein dimer subunits.

**Fig. 1:**
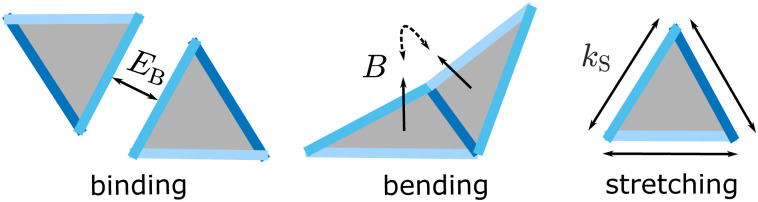
Schematic of the model. The Hamiltonian includes terms that represent edge stretching, monomer-monomer binding, and bending. Each of the three monomer edges is a different type, and only pairs of edges with the same type can bind. In this work, all edge types have the same binding energy. Each edge type *i* has a different preferred (‘ideal’) dihedral angle, *θ*^(*m*,*n*)^_id,*i*_, the set of which determine the target structure (*m*, *n*). The energetic cost of deviations from preferred edge lengths and the dihedral angles are controlled by the stretching and bending moduli, *k*_S_ and *B*.

The Hamiltonian is1
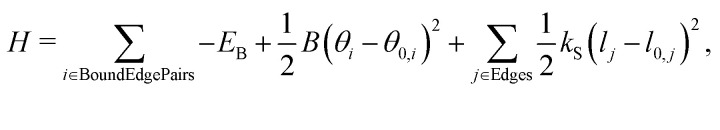
with *E*_B_ as the monomer-monomer binding energy (set as a positive constant); *θ*_*i*_ and *θ*_0,*i*_ as the instantaneous and preferred dihedral angle between two monomers bound at a common edge *i*, *B* as the bending modulus; *l*_*j*_ and *l*_0,*j*_ as respectively the instantaneous and stress-free lengths of an edge *j*; and *k*_S_ as the stretching modulus. The three monomer edges are inequivalent, and setting *θ*_0_ at each of the monomer edges defines the ground state (target) tubule geometry (Fig. S1, ESI†). For simplicity, we set *E*_B_ and *l*_0_ to be identical among all three edges of a monomer, and we consider only a single monomer species. Moreover, motivated by the material properties of DNA origami subunits and proteins, we focus here on the limit of thin sheets, in which the bending deformations are much lower in energy than stretching. Therefore, we set *k*_S_ = 200*k*_B_*T*/*l*_0_^2^ throughout this study so that the monomer edges are nearly fixed in length, and we vary the bending modulus as a control parameter (Fig. 1).

We use Monte Carlo moves to relax the structure, including vertex moves to relax structural degrees of freedom, monomer association and dissociation moves to model assembly and disassembly, as well as moves to model internal rearrangement events such as the splitting and merging of cracks within a structure. All the moves guarantee detailed balance (see ESI† Section IX and X for details about the algorithm and the moves). Provided that this set of movements represents the transitions that are relevant for actual tubule assembly, with approximately correct relative rates for the different moves, the Monte Carlo trajectories can be qualitatively mapped onto the system dynamics. This mapping can be tested by comparing simulation results against experimental observations of tubule assembly kinetics and the structural ensemble of assembled tubules. The edge fusion and fission moves, which respectively bind two free edges on the structure boundary or split two edges that are already bound, are particularly important for closure/reopening of the tubule structure. We show below that the rate of tubule closure relative to its growth can significantly affect the assembly pathways. Therefore, we define a control parameter – the *edge fusion rate f*_fusion_, as the ratio between the attempt frequency of edge fusion/fission moves and the unit timescale (which is set to the frequency of vertex moves).

To determine well-defined steady-state distributions, we evolve the system in the grand canonical ensemble, in which the assembling structure exchanges monomers with a bath at a fixed chemical potential *μ*. This situation approximately describes tubule distributions at a point in a reaction with a corresponding free monomer concentration *c*_0_ = *c*_SS _exp(*μ*/*k*_B_*T*) with *c*_SS_ the standard state concentration. We set *μ* = −3*k*_B_*T* throughout this study. For the purposes of comparing our results with the DNA origami tubule assembly experiments,^9^ we use the same standard state concentration as specified in that work, *c*_SS_ ≈ 10 μM (corresponding to approximately 100% monomer volume fraction). This results in a bath concentration of *c*_0_ ≈ 500 nM. See Section II C for a discussion of how our simulated system compares to the experiments and our rationale for parameter choices.

To model assembly from a dilute system of monomers, for which binding between different tubules is negligible, simulations are restricted to have only one structure within the simulation box. The initial condition for each dynamical assembly simulation is one monomer in the simulation box, which then assembles (and disassembles) through association (and dissociation) of monomers through exchanges with the bath. Since monomers can only associate to an existing structure, and only single monomer associaton/dissociation is allowed, the system is guaranteed to maintain only one structure. For thermodynamic integration, the system is initialized from, and restricted to, a closed tubule lattice (see Section III A and VII B, ESI†).

### B. Simulation results

#### Assembly trajectories

As expected from generic models of tubule or filament assembly,^15^ assembly in our model requires that the subunit concentration and binding affinity exceed threshold values. Below these values, the mixing entropy of free monomers out-competes the interactions stabilizing tubule formation, and equilibrium is dominated by monomers. For the subunit concentration that we focus on (determined by *μ* = −3*k*_B_*T*), we find that the critical binding affinity corresponds to *E*_B_ ≈ 4.5*k*_B_*T*. This observation is supported by our thermodynamic integration results (described in Section III A and VII B, ESI†). As shown in ESI† Section VII B, with *E*_B_ = 4.5*k*_B_*T*, the bulk energy density of an assembled tubule is about −3*k*_B_*T*, and thus equal to the bath chemical potential.

**Fig. 2:**
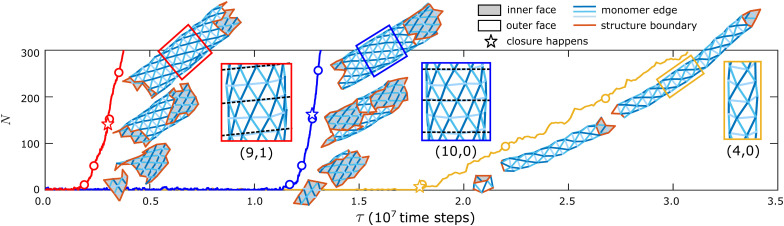
Simulation trajectories showing the number of monomers *N* in a self-assembled structure as a function of elapsed time *τ* (number of Monte Carlo sweeps). Snapshots show the configurations at indicated times. Dashed lines in the zoomed-in snapshots label the direction of the edge that is the most perpendicular to the longitudinal direction. The assembled tubule geometries are: (9,1) for the red trajectory, (10,0) for the blue trajectory, and (4,0) for the yellow trajectory. The red and blue trajectories have the same target tubule geometry of (10,0) and the same binding energy of *E*_B_ = 6.0*k*_B_*T*. The target tubule geometry for the yellow trajectory is (5,0) and the binding energy is *E*_B_ = 5.0*k*_B_*T*. Other simulation parameters are the same for all the trajectories: *B* = 20*k*_B_*T*, *f*_fusion_ = 10^−3^.

Above the threshold binding affinity, the Monte Carlo trajectories exhibit a rich dynamics which proceeds through a series of stages, including nucleation, closure, and growth. Fig. 2 shows snapshots from example simulation trajectories at three different parameter sets. During assembly with a large target tubule diameter (red and blue curves), after an initial period of transient assembly and disassembly, the structure surpasses the critical nucleus size (approximately 5 monomers for these conditions) and grows steadily as a curved two-dimensional sheet. Eventually, the boundary edges at opposite sides of the curling sheet begin to touch, and the edges bind. We denote the first such binding event as the point of tubule *closure*. After closure, the tubule geometry is highly stable and the structure undergoes steady growth from both ends.

Even though the red and blue trajectories have the same target structure, they assemble different tubule geometries, denoted by different pairs of integer numbers (*m*, *n*) based on the convention from carbon nanotubes.^38^ Representing a tubule as a curled triangular lattice that closes upon itself, the index *m* gives the number of lattice sites on one turn around the helix, while *n* gives the number of lattice sites in the orthogonal direction (along the long axis of the tubule) (see Fig. S1 for a schematic of the naming convention and Section I A, ESI† for a detailed description of the notation). In the trajectory with a small target width (yellow line), tubule closure corresponds to the formation of the critical nucleus, after which the tubule undergoes steady growth.

#### Tubule geometry distributions

We performed simulations over a wide range of parameter values to learn how the tubule morphologies arising from dynamical trajectories depend on the relevant physical parameters, such as the bending modulus *B*, the diameter of the target tubule geometry *D*_0_, and the fusion rate *f*_fusion_, which influences the closure kinetics. We measured the distribution of tubule geometries at the end of each simulation. Simulations were performed until the length of the structure *L* grew to approximately three times the tubule circumference, since the geometry distribution is stable by this point (no geometry fluctuations occur beyond this size). We estimated the distributions from 1000 independent trials at each parameter set.

**Fig. 3:**
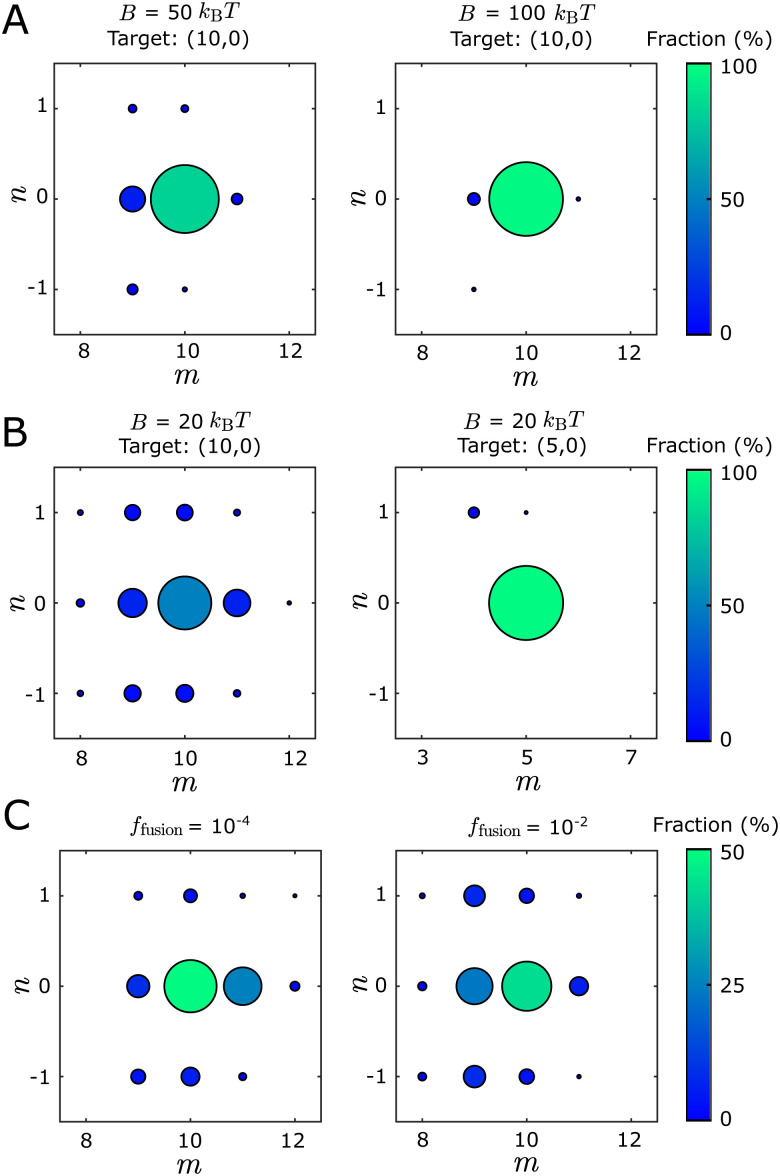
Tubule geometry distributions from Monte Carlo assembly trajectories depend on the bending modulus *B*, target tubule geometry, and fusion rate *f*_fusion_. The color and size of each circle indicate the fraction of the corresponding tubule geometry within the defect-free population. (A) Geometry distributions for *B* = 50*k*_B_*T* (left) and *B* = 100*k*_B_*T* (right) with *E*_B_ = 6*k*_B_*T*, *f*_fusion_ = 10^−3^, and target tubule geometry (10,0). The fraction of defect-free tubules is about 97% for both cases. (B) Geometry distributions for different target geometries with *B* = 20*k*_B_*T*, *E*_B_ = 6*k*_B_*T*, and *f*_fusion_ = 10^−3^. The fraction of defect-free tubules is about 88% for both cases. (C) Geometry distributions for indicated values of *f*_fusion_ with *B* = 20*k*_B_*T*, *E*_B_ = 6*k*_B_*T*, and target tubule geometry (10,0). As *f*_fusion_ decreases from 10^−2^ to 10^−4^, the fraction of defect-free tubules decreases from 88% to 52%, while the fraction of open structures increases from 0 to 45%. Each distribution in (A–C) is estimated from 1000 independent simulation trajectories.

We find that tubule structures with different geometries, as well as structures that fail to close, can assemble in the dynamical simulations under the same set of parameter values. We classify the self-assembly outcomes into three categories: *defect-free* tubules, *defective* tubules, and *open structures*. A tubule is *defective* if part of the structure fails to close or multiple tubule geometries are locally identified within the same structure (see Fig. S3 and Section III A, ESI† for identification details). *Open structures* arise when nonuniform curvature causes opposite boundary edges to ‘miss’ the opportunity to bind to each other, leading to a spiral structure that resembles a toilet paper roll (Fig. S3, ESI†). The fraction of defective tubules increases as the bending modulus *B* decreases or the target diameter *D*_0_ increases (Fig. 3). The fraction of defective and open structures increases with the binding energy (Fig. S10, ESI†). To avoid conditions under which defective structures are too prevalent, for all results in the main text we set the binding energy to *E*_B_ = 6*k*_B_*T* and the chemical potential to *μ* = − 3*k*_B_*T*, so that assembly is sufficiently reversible to allow monomer detachment and annealing.^39–42^ With these parameters, the fraction of defective tubes is generally below 30%.

The geometry distributions of defect-free tubules depend on the control parameters. Fig. 3 shows the distributions of assembled tubules for different bending moduli, target diameters, and fusion rates. The size and color of the circular symbols represent the fraction of different tubule geometries within the defect-free population. Only tubule geometries with populations ≥1% are labeled in the plot. Fig. 3(A) shows tubule geometry distributions for two bending moduli *B*, with other parameters fixed. As *B* increases, the fraction of the target tubule (10,0) increases while the fraction of the off-target tubules decreases. This is consistent with thermodynamics, since the deviations of dihedral angles required for off-target geometries increase in energy with *B*. Fig. 3(B) compares the distributions for two different target geometries. As the diameter of the target geometry *D*_0_ increases, the fraction of the target geometry decreases while the fraction and variety of observed off-target geometries increases. This result is also consistent with thermodynamics, since the difference of the ideal dihedral angles between the target state and neighboring tubule geometries is smaller for larger *D*_0_ (Fig. S2, ESI†). Therefore, with the same extent of dihedral angle fluctuation, the number of accessible off-target states increases as *D*_0_ increases. Similar results were described in ref. 43.

As shown in Fig. S10 (ESI†), the tubule geometry distribution does not significantly change for binding energies in the range *E*_B_ ∈ [5,6.5] *k*_B_*T*, suggesting that there is at most a weak dependence on binding energies for the regime we focus on. We discuss this result further in Section III H, ESI.†

Interestingly, even for the same Hamiltonian (in which *B* and *D*_0_ are fixed), changing the edge fusion rate *f*_fusion_ changes the skew of the geometry distribution. Fig. 3(C) shows that as *f*_fusion_ increases from 10^−4^ to 10^−2^, the geometry distribution changes from skewing above to skewing below the target geometry (10,0). Meanwhile, the proportion of open structures increases from 0 to around 45% as *f*_fusion_ decreases from 10^−2^ to 10^−4^ (Fig. S7, ESI†). This observation reflects the fact that decreasing the closure rate increases the chance that the two edges ‘miss’ each other. As the two edges grow past one another, the size of a curvature fluctuation required to enable closure becomes increasingly unfavorable energetically, and thus more rare. The continued growth of the structure boundary then leads to the spiraling structure described above. See Section III D for further discussion.

### C. Comparison of simulations and experiments

We now compare the results of our dynamical assembly simulations to recent experiments. We find that the morphology distribution of simulated tubules semi-quantitatively agrees with those observed in the experiments,^9^ suggesting that the model incorporates the essential physics of the experiments.

**Fig. 4:**
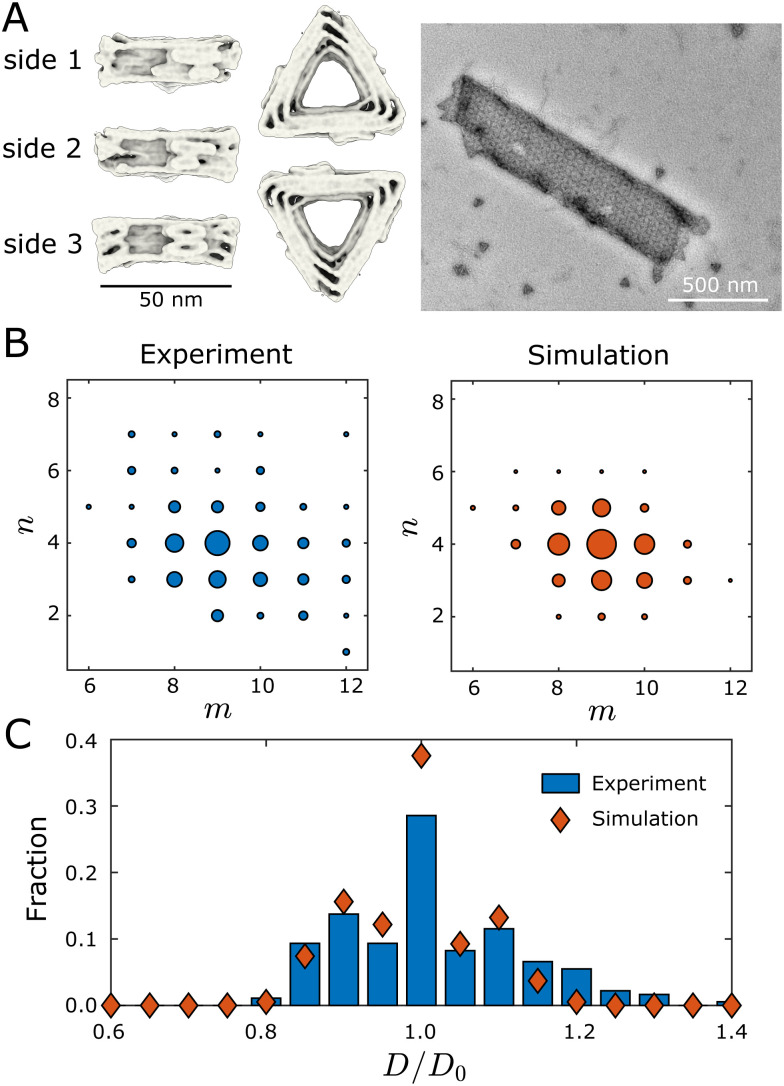
Comparing the geometry distributions of tubules assembled in simulations and experiments. (A) Cryogenic electron microscopy reconstructions of a DNA origami monomer, and a transmission electron microscopy image of an assembled tubule. The left panel shows the monomer under different views. Two monomers bind along their edges through shape-complementary interactions driven by blunt-end DNA base stacking. The right panel shows an assembled (9,4) tubule. Images in (A) were provided by authors of ref. 9. (B) Tubule geometry distributions measured from experiments^9^ and simulations. The size of each circle indicates the fraction of the corresponding tubule geometry within the defect-free population. Each simulation data point is estimated from 1000 independent dynamical MC trajectories that are initialized from a single monomer. (C) Comparing tubule width distributions between experiments (blue bars) and simulations (red symbols). *D*_0_ is the diameter of the ideal (9,4) tubule. Simulation parameters: the target geometry is (9,4), *E*_B_ = 6.0*k*_B_*T*, *B* = 10*k*_B_*T*, and *f*_fusion_ = 10^−3^.

Hayakawa *et al.*^9^ designed triangular monomers from DNA origami that self-assemble into helical tubules (Fig. 4). The monomers interact with each other along their edges through shape-complementary interactions driven by blunt-end DNA base stacking.^8^ The interactions are specific—each monomer edge interacts only with the same edge type on a neighboring subunit. The bevel angles of the edges of each monomer {*θ*^0^_id,*i*_} determine the preferred dihedral angles, which, in turn, set the preferred curvatures of the assembly.

The data set against which we compare our simulation results is obtained from an experimental system that resulted in a most probable tubule geometry of (9,4). Since a key unknown parameter from the experiments is the bending modulus *B*, we performed simulations with a target geometry of (9,4) at four values of the bending modulus: *B* ∈ {5,10,15,20} *k*_B_*T*. All other parameters were fixed to their default values (see Section II). We found that assembled structures were highly defective for *B* = 5*k*_B_*T*. For *B* ≥ 10*k*_B_*T* the majority of tubules were well-formed, with distributions peaked around the target geometry of (9,4). The width of the distribution becomes progressively narrower with increasing *B*, as described in Section II B.

We found that a value of *B* = 10*k*_B_*T* resulted in a geometry distribution of assembled tubules that closely resembles the distribution observed in the experiments (Fig. 4(B)). To facilitate comparison between the two distributions, Fig. 4(C) plots the fraction of different tubule geometries against the diameter of the tubules, where *D*_0_ is the diameter of the (9,4) tubule geometry. Although the simulation distribution is slightly narrower than the experiment, we observe that the distributions match fairly closely, especially considering that we have not quantitatively optimized *B*. Results for the other simulated values of *B* are shown in Fig. S11, ESI.†

The comparison between simulations and experimental results in Fig. 4(B) and Fig. S11 (ESI†) suggests several important qualitative conclusions: (1) the simple model considered here produces results which are semi-quantitatively consistent with those observed in the experiments. As we will show below, these results can only be explained through a combination of kinetic and thermodynamic effects, which suggests that the highly simplified dynamics of our model captures the most relevant physics. (2) It was not possible to directly estimate the bending modulus within the experiments. The simulation results suggest a bending modulus on the order of 10*k*_B_*T*, which is comparable to that of a lipid bilayer membrane (*B*/*k*_B_*T* ∼ 10–20).^44,45^ This computational result could be tested in future experiments that measure the distribution of angular fluctuations between monomers. (3) Given the qualitative agreement between the computational and experimental results, the simulations can provide a predictive guide for future experiments. We note that a definitive comparison of our simulation results to the experiments, and a precise estimate of the experimental bending modulus, will require additional experimental data sets. In the subsequent sections, we use the simulations and simple models to understand the effect of relevant control parameters on the morphology distributions of assembled tubules.

#### Comparison between simulated systems and the experimental conditions in ref. 9

We have chosen parameters that ensure the simulations are qualitatively similar to the motivating experiments while maintaining computational tractability. In particular, we focus on values that place the simulations in the same assembly regime as observed in the experiments, while enabling sampling a statistically significant ensemble of assembly trajectories. We summarize these parameters and their correspondence to the experiments here.

In Hayakawa *et al.*,^9^ the total monomer concentration is *c*_0_ ≈ 10 nM. If we set our standard state volume to that used in Hayakawa *et al.*, *c*_SS_ = 1/*ν*_monomer_*N*_A_ ≈ 10 M, with *v*_monomer_ the volume of a monomer and *N*_A_ Avogadro's number, then our chemical potential value *μ* = − 3*k*_B_*T* corresponds to a bath concentration of *c*_0_ ≈ 500 nM.

We use this larger concentration because it allows assembly dynamics to occur on faster timescales compared to the experimental concentration, thus making our dynamical simulations more computationally tractable. The higher concentration causes association to occur on shorter timescales, and, to maintain the same assembly regime as in the experiments, the binding energies can be somewhat smaller, making unbinding occur on shorter timescales. In particular, for *μ* = − 3*k*_B_*T*, the threshold binding energy for assembly is *E*_B_ ≈ 4.5*k*_B_*T* (see Section II B and VII B, ESI†). For our dynamical simulations to be in the experimentally relevant regime, they require binding energies that are somewhat larger than the threshold energy, so that nucleation occurs within computationally accessible timescales (and similarly experimentally accessible timescales), which corresponds to the value we focus on, *E*_B_ = 6.0*k*_B_*T*. At this binding energy, the probability for a pair of fused edges to undergo fission becomes sufficiently small that the closure event is nearly irreversible, but assembly still involves a significant nucleation barrier. Both conditions are consistent with experimental observations. Recall also that our simulation algorithm enforces the dilute assembly regime by construction, consistent with the low experimental concentration. Moreover, the net bulk free energy density of assembled tubules in the simulations, Δ*ε* ≈ − 3*E*_B_/2 − *μ* = − 6*k*_B_*T* is close to the value at early stages of the experiments (before free monomers are significantly depleted): with *c*_0_ = 10 M the initial chemical potential is *μ* ≈ − 7*k*_B_*T*, while the binding energy was estimated as *E*_B_ ≈ 9*k*_B_*T*, resulting in Δ*ε* ≈ − 6.5*k*_B_*T*.

Other differences between the simulations and experiments are as follows. The simulations are performed in the grand canonical ensemble and thus have a constant bulk monomer concentration. In contrast, the experiments are in the canonical ensemble (fixed total monomer concentration). Thus, the bulk monomer concentration is depleted as assembly occurs, so tubules that nucleate at later times in the experiment assemble at lower chemical potentials. Tubule morphology distributions are measured over all times, and thus average over these differences in bulk concentration. However, based on the observation that the width distribution of well-formed tubules does not depend sensitively on binding energy in this parameter regime (Fig. S10, ESI†), we expect that it is also insensitive to the chemical potential. In addition, the tubules in the experiments have an exponential distribution of lengths (as expected for the canonical ensemble). The experiments are performed for one week, by which time the longest tubule structure is about 2 μm, which is roughly 10 times its diameter. In the simulations, we analyze all tubules after they have grown to a length of 10 times the tubule diameter. However, this difference does not affect the results, since, as we show in this work, the morphology distribution is set at the time of closure and is thus roughly independent of tubule length.

#### Controlling the monomer–monomer binding affinity

The experiments show that the monomer–monomer binding affinity increases with increasing salt concentration,^8,9^ likely due to screening of electrostatic repulsions between monomers. Our binding affinity parameter *E*_B_ thus qualitatively maps to the salt concentration used in the experiments. However, the affinity increases much more strongly with magnesium compared to sodium than would be predicted by simple screening, suggesting specific interactions with magnesium. Therefore, we have not attempted a quantitative mapping between *E*_B_ and experimental parameters.

## III. Theoretical models

To determine whether kinetic effects influence the observed geometry distributions, we compute the equilibrium tubule geometry distribution and compare it against those observed in simulations. We first perform the calculation accounting for the discrete tubule geometries allowed by the finite monomer size, and then we simplify the calculation by adopting the continuum limit.

### A. Discrete equilibrium model

Motivated by the high rigidity of DNA origami subunits,^8,9^ we focus on the regime of high stretching modulus in this work, so that the relative edge length fluctuations are small, *k*_B_*T*/*k*_S_*l*_0_^2^ ≪ 1. Thus, in the following calculation we assume that the curvature within a tubule is uniform, and for an assembled geometry (*m*, *n*) all the dihedral angles are approximately equal to their ideal value *θ*^(*m*,*n*)^_id,*i*_, with *i* ∈ 1, 2, 3 as the indices of the three sides of a subunit. We denote the ideal angles for the target geometry as *θ*^0^_id,*i*_. The free energy per monomer *g*^(*m*,*n*)^_*L*_ in a tubule geometry (*m*, *n*) with length *L* is then approximately given by2
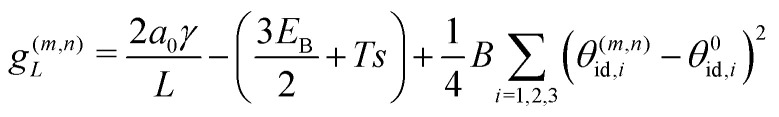
in which *a*_0_ is the area of a monomer, *γ* is the line tension accounting for unsatisfied interactions at the two tubule boundaries, *E*_B_ is the binding energy, *B* is the bending modulus, *T* is the temperature, and *s* is the per-monomer entropy. The bending energy term is reduced by a factor of two compared with eqn (1) because it is shared between two neighboring monomers. The equilibrium probability *P*^(*m*,*n*)^ to assemble the tubule geometry (*m*, *n*) with length *L* is given by

3 *P*^(*m*,*n*)^_*L*_ ∝ exp [−*βN*(*g*^(*m*,*n*)^_*L*_ − *μ*)],

where *N* is the number of monomers in the structure and *μ* is the chemical potential. In the grand canonical ensemble *μ* is equal to the bath chemical potential, while in the canonical ensemble (conserved total monomer concentration) *μ* = *k*_B_*T* ln (*c*_0_/*c*_SS_) with *c*_0_ the concentration of free monomers, with *c*_SS_ the standard state concentration.

We consider the large *L* limit, in which the contribution from the line tension can be ignored, so the free energy per monomer becomes independent of length and will be denoted as *g*^(*m*,*n*)^. Further, at equilibrium, the free energy per monomer of the geometry that minimizes the free energy (in this case the target geometry) is approximately equal to the chemical potential *μ*.^15,46^ Since the bending energy of the target geometry is zero, the equilibrium chemical potential is given by 
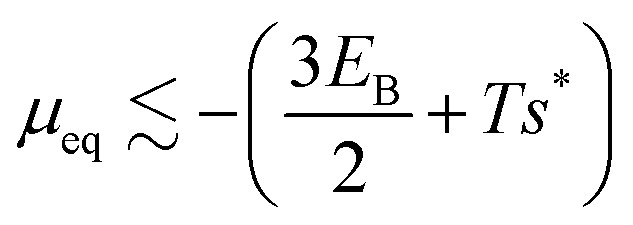
, where s* is the entropy per monomer of the target structure. Assuming the entropy is roughly independent of geometry, the probability distribution is then dominated by the bending energy, resulting in4
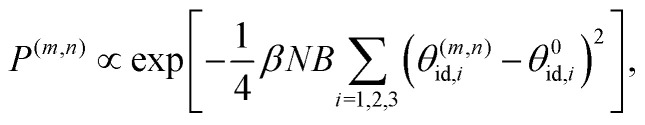
which shows that the probability to assemble an off-target structure decreases exponentially as the bending modulus *B* increases.

To test this analysis, we used an adapted thermodynamic integration algorithm to compute the free energy for different tubule geometries *g*^(*m*,*n*)^_*L*_. In brief, the algorithm evaluates the free energy change for each geometry along a thermodynamic pathway that gradually transforms the Hamiltonian of the system from a reference state (an Einstein solid with the same number of vertices) to our computation model (eqn (1)). We find that the measured free energy difference between different tubule geometries closely agrees with the bending energy difference, confirming the validity of the simplifications described above. See Fig. S17 and Section VII, ESI† for details about free energy computations and the comparison to the bending energy.

### B. Continuum equilibrium model

To obtain an approximate analytical expression for the tubule width distribution, we adopt the continuum limit and neglect the presence of defects. In this limit, the bending energy as a function of tubule diameter *D* is given by the Helfrich energy^47^5
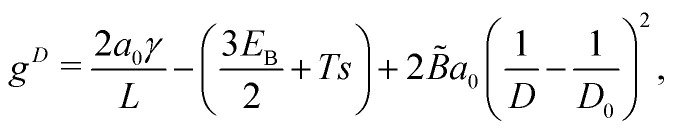
with *D*_0_ as the diameter of the target structure and *B̃* as the effective bending modulus in the continuum limit. The continuum bending modulus is related to the bending modulus *B* of the discrete model by 
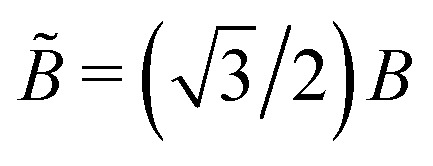
.^30^

We evaluate the equilibrium width fluctuations of the closed tubules 
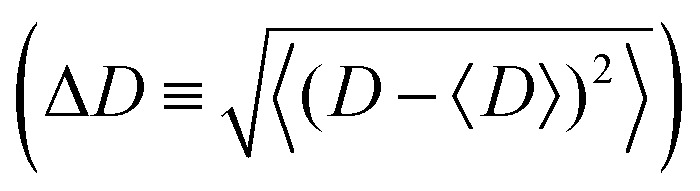
 as a function of their length *L*. By performing analogous simplifications to the discrete model (see Section II, ESI†), we obtain:6
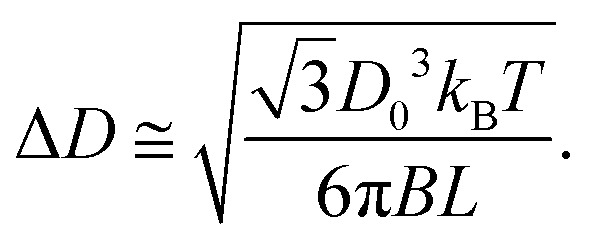


Eqn (6) shows that the relative equilibrium width fluctuations decrease with bending modulus, but increase with target diameter as Δ*D*/*D*_0_ ∼ *D*_0_^1/2^, as found for spherical curvature-controlled capsids.^15^ However, a key difference for tubules is that the equilibrium fluctuations become negligible for tubules with large aspect ratios *L* ≫ *D*. Thus, the observations from simulations and experiments of appreciable width fluctuations in large-aspect ratio tubules indicate that kinetic effects are important in determining the polymorphism.

### C. Comparing simulation results against equilibrium width distributions

**Fig. 5:**
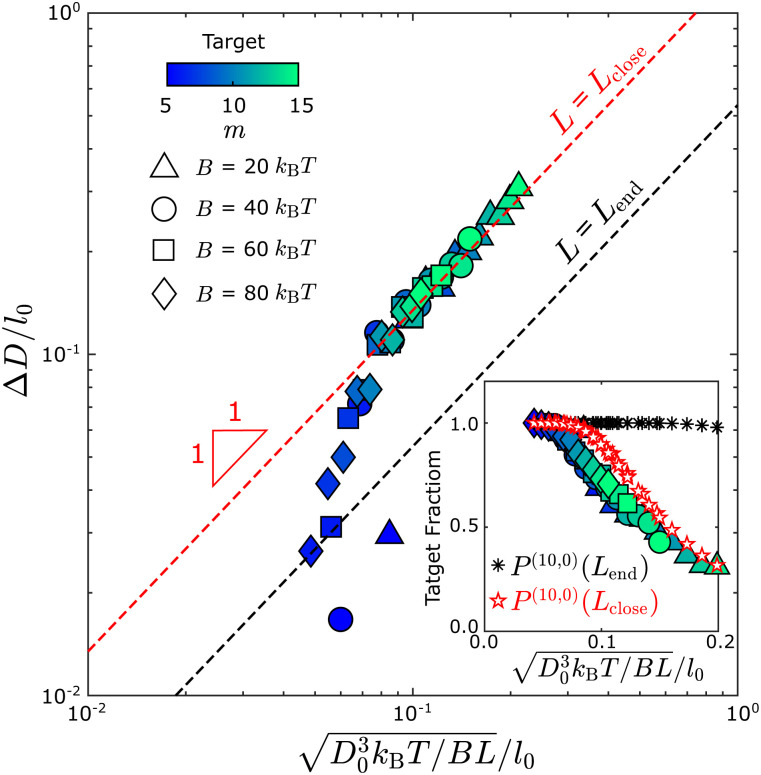
Comparing the tubule geometry distribution from Monte Carlo assembly trajectories with the equilibrium theory shows that tubule closure fixes tubule geometries out of equilibrium. Tubule width fluctuations Δ*D* measured from simulations at different parameter sets, plotted according to the scaling from equilibrium theory (eqn (6)). The dashed black line shows the equilibrium result for the tubule length at the end of the simulation (with *L* = *L*_end_ in eqn (6)), while the dashed red line shows the expected result if the geometry is quenched at the point of closure (with *L* = *L*_close_ in eqn (6)). Different symbols represent different bending modulus values *B*, and the color shows the first lattice number *m* of the target tubule geometry (*m*, *n*); all structures in this dataset have *n*= 0. The inset shows an analogous comparison for the fraction of tubules within the defect-free population that have the target geometry. The black asterisk symbols show the discrete model prediction (eqn (4) with *L* = *L*_end_) and the red pentagon symbols show the discrete model prediction with *L* = *L*_close_. Other simulation parameters: *E*_B_ = 6*k*_B_*T* and *f*_fusion_ = 10^−3^.

By comparing the simulation and equilibrium computation, we find that tubule geometry distributions from the dynamical simulations have larger variances than predicted by the equilibrium models. Fig. 5 compares the width fluctuations Δ*D* measured in the simulations to the scaling law (eqn (6)) from the equilibrium computation for different values of the bending modulus and target geometry. In general, we see that the distributions observed in simulations have larger variances than the equilibrium results. Importantly, the observed Δ*D* collapse to the equilibrium scaling with respect to the target diameter *D*_0_ and bending modulus *B*, but not at the tubule length at which the geometry measurements are performed (*L*_end_ ∼ 3π *D*_0_, black dashed line in Fig. 5). The measured diameter fluctuations are much larger than the equilibrium value. Instead, the fluctuations are roughly consistent with the equilibrium prediction for the smaller value of *L*_close_ ∼ 1.5 *D*_0_ at which the tubules closed. Indeed, the results match the equilibrium prediction with *L* = *L*_close_ for all parameter values except Δ*D*/*l*_0_ ≲ 0.5; below this threshold the fluctuations are smaller than the discrete monomer size and the continuum approximation breaks down. Note that similar results were observed for the same computational model in ref. 43 and were shown to be consistent with experiments on DNA origami subunits assembling the tubules in ref. 9. A detailed description of how we measure the tubule diameter and the closure size is given in the ESI† (Fig. S4 and S5).

The fact that the fluctuations are consistent with the equilibrium prediction, but at the smaller length *L*_close_, indicates that the geometry distribution is *kinetically* controlled. This conclusion is consistent with the observation from simulations that the geometry rarely changes once a tubule closes, which can be understood from the fact that, after closure, all monomers have their maximum number of bonds except those at the two tubule ends. Rearrangement of the tubule geometry requires breaking a significant number of bonds, and thus overcoming a large free energy barrier. Note that the substitution of *L*_close_ into eqn (6) amounts to a quasi-equilibrium assumption: because assembly occurs near equilibrium for the parameters considered in Fig. 5, the tubule geometry distribution at the time of closure is nearly consistent with the equilibrium distribution at the corresponding tubule length *L*_close_. However, this condition breaks down for larger values of the edge fusion rate *f*_fusion_ as discussed next.

**Fig. 6:**
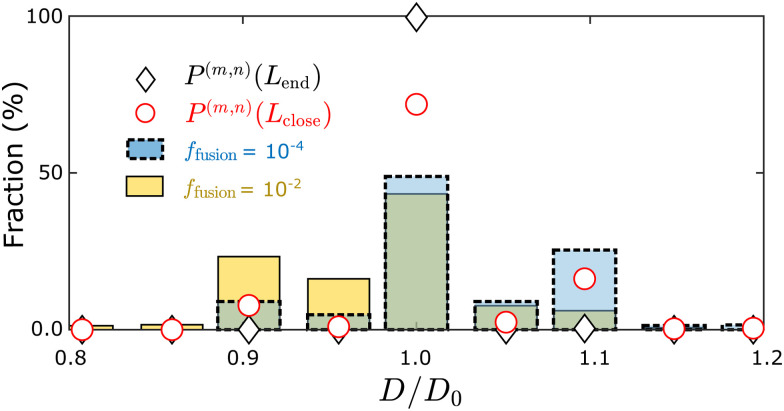
Comparing measured tubule width distributions to the discrete equilibrium model (eqn (4)). Bars are the simulation results with indicated values of *f*_fusion_, while symbols represent the equilibrium results, with tubule length at the simulation endpoint or at closure respectively. Other simulation parameters: *B* = 20*k*_B_*T*, *E*_B_ = 6*k*_B_*T*, and the target tubule geometry is (10,0).

We also compared the fraction of each tubule geometry (*m*, *n*) predicted by the discrete model against the simulation results, which indicated a similar trend as for the continuum model: The distribution computed using *L*_close_ is much closer to the simulation results as compared with using *L*_end_ (Fig. 6), in terms of the width distribution and the yield of the target geometry (Inset of Fig. 5). Here, we replot the distribution in Fig. 3(C) against the diameter of the assembled tubule geometries (bars in Fig. 6). However, as *f*_fusion_ increases from 10^−4^ to 10^−2^, the skewness of the tubule width distribution changes from below to above *D*_0_. The equilibrium computation at *L*_close_ does not predict the change in skewness resulting from the change in the assembly kinetics.

This result shows that the simple picture based on a quasi-equilibrium morphology distribution at *L*_close_ does not capture all kinetic effects that control the tubule morphology distribution. In Section III D we develop a model that accounts for these additional dynamical influences.

### D. Kinetic model for tubule geometry distributions

The results shown thus far suggest that factors affecting the size and geometry of the tubule at the moment of closure are the key determinants of the observed steady-state geometry distribution. In this section, we develop a discrete model that incorporates both the kinetics and thermodynamics of the system and we show that it semi-quantitatively describes the simulation results. We present an analogous continuum model in Section V C, ESI.†

To simplify the analysis, we focus on parameters for which the critical nucleus size is small compared to the closure size, which covers most of the parameter space that we consider in this work (see Fig. 2). Therefore, in the model we assume that nucleation occurs well before closure, and thus the two processes are independent. In a future work, we will extend the model to account for the case when the closure and critical nucleus sizes are comparable, and thus the two processes are coupled.

We consider the structure before closure as a circular disk that is bent to have the stress-free curvature of the target tubule (see Fig. S12, ESI† for a schematic of the model). The size *N* of the disc grows with a rate *k*_grow_ that is proportional to its boundary length:7
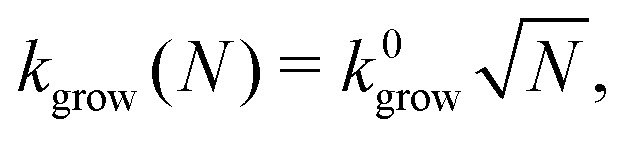
where *k*^0^_grow_ is a factor that depends on *B* and *E*_B_ (see Fig. S8 and Section III F, ESI† for details about growth rate measurements). To simplify the model, we ignore the stochasticity in subunit association by assuming that *N* increases by one subunit at regular time intervals given by Δ*t* = 1/*k*_grow_.

While remaining at a size *N*, the open structure also attempts to close with a rate *k*_close_. Once the structure closes, we assume that it does not reopen. Allowing for a finite reopening probability is straightforward, but has a negligible effect on the results for the parameters that we focus on because reopening is rare and/or transient. We assume *k*_close_ decreases exponentially with the free energy barrier to closure Δ*G*_close_, which arises primarily from the bending elastic energy due to the difference in the curvature of the closed structure and the stress-free structure. Section III G, ESI† presents estimates and measurements of Δ*G*_close_.

At a given size *N*, the rate *k*^(*m*,*n*)^_close_ of closing into a structure (*m*, *n*) is then approximated by8
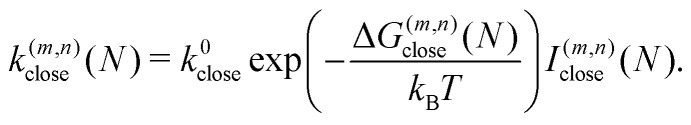
Here *k*^0^_close_ is the closure attempt rate (*i.e.* the rate in the absence of a barrier), and *I*^(*m*,*n*)^_close_ is a function that indicates whether a particular structure (*m*, *n*) is geometrically compatible with closure at size *N*: *I*^(*m*,*n*)^_close_ = 1 if it is compatible and *I*^(*m*,*n*)^_close_ = 0 if it is incompatible (see Section V B, ESI† for details about the determination of *I*^(*m*,*n*)^_close_ (*N*)). Assuming that shape fluctuations are fast in comparison to the net growth timescale, the net closure rate *k̃*_close_ for a disk with size *N* is then given by a sum over all accessible geometries as9
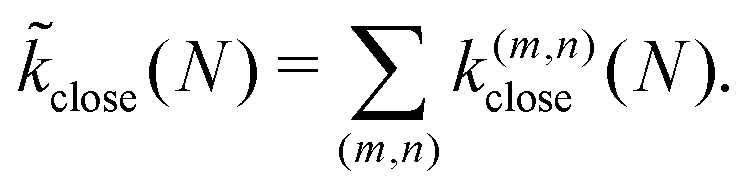


Finally, we evaluate the closure probability as a function of time. To simplify the calculation, we assume that the structure is larger than the critical nucleus size, and that closure is a rare event in comparison to growth. In the absence of closure, the time at which a structure first grows to size *N* is thus 
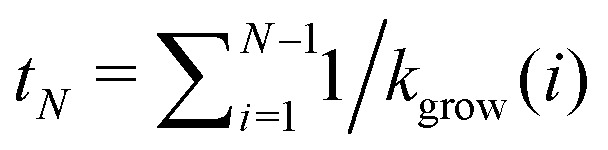
, and the probability that such a structure stays open for an additional time *δt* < *t*_*N*+1_ − *t*_*N*_ is

10 *P*_open_(*t* + *δt*, *N*) = *P*_open_(*t*_*N*_,*N*)exp [−*k̃*_close_(*N*)*δt*].

By summing over smaller sizes, we can compute the probability that a structure has stayed open until size *N* as11
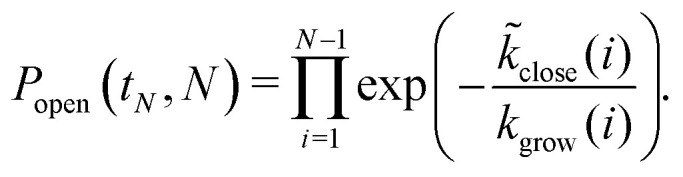


The probability for the structure to close at size *N* is then given by12
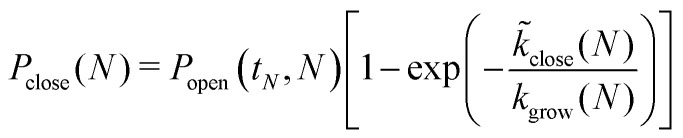


The probability to assemble a geometry (*m*, *n*) is then computed by summing over all sizes *N* that can close to (*m*, *n*)13
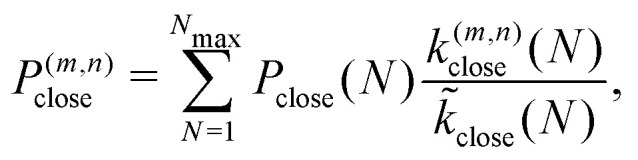
where the second term on the right-hand side is the conditional probability for assembling the geometry (*m*, *n*), given that the structure closes at size *N*. Eqn (13) shows that the ratio of growth to closure rates, which is a kinetic factor, can significantly affect the tubule geometry distribution. Next, we will compare these predictions against the dynamical simulation results from Section II B.

### E. Testing the kinetic model predictions

The simple kinetic model predicts the tubule geometry distribution and *yield* of the target geometry over a wide range of parameter space of the bending modulus *B* and the effective closure rate (normalized by the net growth rate, log (*k*^0^_close_/*k*^0^_grow_)). We define the yield as the fraction of a specific defect-free tubule geometry (*m*, *n*) within the entire population (including the unclosed structures and the defective tubules), and *k*^0^_close_ is defined as the rate for an isotropic open structure to close and form the target geometry. In the simulations, the effective closure rate log(*k*^0^_close_/*k*^0^_grow_) is controlled by the parameter *f*_fusion_, and we measured log(*k*^0^_close_/*k*^0^_grow_) from simulation trajectories as described in Section III F and G, ESI.†

**Fig. 7:**
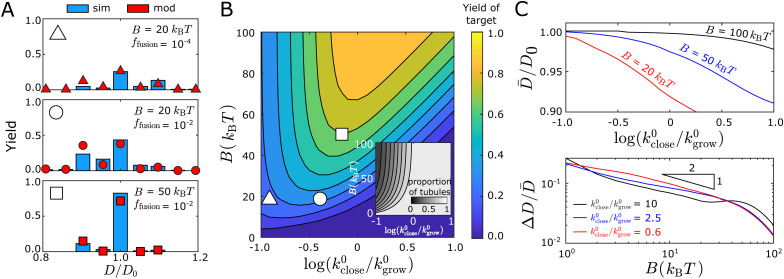
The kinetic model captures the geometry distributions observed in simulations. (A) Comparison of the tubule width distribution between the kinetic model (red symbols) and simulation results (blue bars) for three representative parameter sets. The triangle, circle, or square symbol at the top left of each panel indicates its corresponding location in the parameter space for the plot shown in (B). The yield is defined as the fraction of a tubule geometry assembled within the *entire* population of the assembled structures, including the structures that do not close. (B) Color map showing the yield of the target tubule geometry (*P*^(10,0)^_close_ defined in eqn (13)) predicted by the kinetic model as a function of the bending modulus *B* and the normalized closure rate *k*^0^_close_/*k*^0^_grow_ (shown on a log scale). The inset shows the fraction of closed tubules predicted by the model (
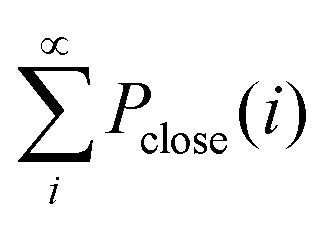
, *P*_close_ (*i*) is defined in eqn (12)).(C) Kinetic model predictions for the mean (*D̄*, top panel) and coefficient of variation (Δ*D*/*D̄*, bottom panel) with respect to the normalized closure rate and bending modulus. In all cases the target geometry is (10,0).

The kinetic model accurately predicts the detailed distribution of defect-free tubule geometries, as well as the fraction of structures that fail to close in the dynamical simulations. Comparisons between the kinetic model and the simulation results are shown for three representative parameter sets in Fig. 7(A). Starting from the top panel, we reduce *f*_fusion_ by 100 × at fixed *B* (middle panel), which does not significantly change the spread of the distribution of closed tubules, but changes the skew from wider than targeted to narrower than targeted. More significantly, the yield of the target geometry decreases from 40% to 28% while the fraction of the target geometry within the defect-free population does not significantly change. This observation is because the proportion of unclosed structures increases from 0 to ∼45% within the entire population (Fig. S7, ESI†). The kinetic model captures this trend.

In the bottom panel, we increase *B* at fixed *f*_fusion_ (relative to the middle panel), which narrows the distribution considerably and shifts the mean toward the target diameter. In particular, a significant fraction of tubules with sizes < *D*_0_ at *B* = 20*k*_B_*T* shifts to the target geometry with *D*_0_ at *B* = 50*k*_B_*T*. This trend reflects a combination of thermodynamic and kinetic effects. Increasing *B* increases the thermodynamic stability of the target geometry relative to competing structures. It also decreases the net growth rate *k*^0^_grow_ because monomer association incurs a greater entropy penalty (since fewer configurations are accessible for binding at higher *B*), which increases the effective closure rate (see Fig. 7(B)) and thus favors smaller structures. However, the thermodynamic effect dominates in this case and shifts the distribution upward toward *D*_0_ (see Fig. S15, ESI† for details).

Fig. 7(B) shows that the yield of the target geometry also depends on a combination of thermodynamic and kinetic factors. The value of the bending modulus *B* sets an upper limit on the yield, while the yield itself changes nonmonotonically with respect to the normalized closure rate at fixed *B*. These trends reflect the fact that the bending rigidity determines the spread of the distribution, while the closure rate mostly influences the mean of the distribution. As noted above, a higher closure rate leads to structures that close earlier and thus shifts the mean toward smaller structures.

The bending rigidity controls both the mean *D̄* and the width fluctuation Δ*D* of the distribution, while the effective closure rate mostly influences *D̄*. Fig. 7(C) shows the mean of the distribution *D̄* (top panel) and the width fluctuation Δ*D* (bottom panel) as functions of the closure rate and the bending modulus. We see that the mean width monotonically decreases with increasing normalized closure rate or decreasing *B*. In contrast, the fluctuations Δ*D*/*D̄* decrease with *B* but depend only weakly on the closure rate. The latter trend is consistent with the qualitative results from the quasi-equilibrium model (Section III C) based on the equilibrium geometry distribution at the time of closure. In particular, the scaling result Δ*D* ∼ *B*^−1/2^ still applies. However, the results for the mean width reflect the fact that the probability for an open structure with size *N* is determined by both the effective closure rate at that size and the time for the structure to remain at size *N*. As shown above, the closure rate increases with *k*^0^_close_ and decreases with *B*, while the time for the structure to remain at a given size decreases with increasing *k*^0^_grow_. The irreversible nature of tubule closure plays a key role in this trend, since the smaller structures always have the opportunity to close before larger sizes. Thus, increasing the closure rate or extending the time at a given size will cause the entire distribution to shift toward smaller widths.

Interestingly, the closure rate does not significantly influence Δ*D*/*D̄*. Although the kinetic effects discussed above change the tubule closure size *N*_close_, they do not significantly change the relative prevalence of different tubule geometries at a given *N*. Thus, as long as the shift of the distribution away from *D*_0_ is not too large, the density of states around the preferred geometry at size *N*_close_ remains comparable to that around the target geometry. This distribution is then essentially fixed once closure occurs.

## IV. Conclusions

In summary, we have used kinetic Monte Carlo simulations and free energy calculations to understand the dynamical assembly of helical tubules. Our simulations reveal how assembly pathways and the resulting tubule morphologies depend on control parameters. The geometry distribution of assembled tubules predicted by the simulations semi-quantitatively matches the distribution observed in experiments on tubules assembled from DNA origami monomers,^9^ suggesting that the model captures the key physics of the experimental system. Further, we show that the simulations provide a useful tool to obtain a first-order estimate of the physical parameters of the experimental system, and can serve as a predictive guide for future experiments.

Comparison of the simulation results with an equilibrium calculation shows that the geometry distribution of assembled tubules depends on a balance between thermodynamic and kinetic effects. While the observed magnitudes of the fluctuations in the tubule width Δ*D* match the equilibrium scaling 
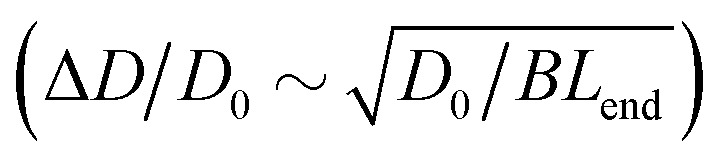
 with respect to bending modulus *B* and preferred diameter *D*_0_, the distribution of assembled tubules is significantly broader and *independent* of length (*L*_end_). This behavior can be explained by the fact that the tubule geometry becomes fixed shortly after an assembling proto-tubule closes upon itself. Closure stabilizes monomer interactions except for those at the two open ends of the tubule, and the topology rearrangements required to significantly change the tubule structure would incur a large free energy barrier. For this reason, the observed geometry distribution fluctuations tend to scale with the tubule length at closure, as *L*^−1/2^_close_. For systems in which closure rates are slow in comparison to growth timescales, the resulting tubule morphology distribution is approximately given by the equilibrium distribution at *L*_close_. However, for systems with faster closure rates (relative to assembly), additional kinetic effects shift the geometry distribution further out of equilibrium. We developed a simple kinetic model which captures these additional effects.

While the computational model used in this work is based on triangular monomers motivated by the DNA origami experiments,^8,9^ experimental and computational systems that assemble tubules from other monomer geometries, such as DNA tiles^14,48^ and wedge-shaped monomers,^23^ exhibit similar polymorphic behaviors as in the DNA origami system. Moreover, the simple kinetic model derived in Section III D does not assume a particular monomer geometry. The high level of agreement between predictions of this model and the computational results suggests that kinetic control of morphology applies generically to helical tubule self-assembly systems in which growth rates are significantly faster than reopening, regardless of the monomer shape.

### Model limitations and outlook

While our kinetic model closely reproduces the computational results over a wide range of parameter space, it is limited to regimes in which tubule closure occurs above the critical nucleus size. In particular, the model assumes a positive net growth of the assembling tubule and thus is limited to the forward-biased growth phase that occurs beyond the critical nucleus size. In a future work, we plan to study the nucleation behavior in detail, and how the assembly kinetics and geometry distribution change when closure occurs before nucleation. In this study we have primarily focused on parameters that lead to well-formed tubules, with a low fraction of defective tubules. However, the simulations provide insights into the factors and mechanisms controlling defect formation. For example, analysis of our simulation trajectories suggests that defective tubules frequently arise when closure happens locally and independently at two or more sites on the boundaries, with geometries that are incompatible with the overall tubule geometry. This mechanism results in a local crack between binding sites, which is unable to anneal unless one of the bound edge-pairs breaks. We expect that the probability of observing defects through this mechanism increases as assembly and closure become less reversible, *via* increasing the binding energy *E*_B_ or the intrinsic closure rate constant *k*^0^_close_, or decreasing the bending modulus *B*. On one hand, the frequency of multiple independent closure events, and thus the probability of observing cracks by this mechanism, increase with the effective closure rate. As described in Section III D, the effective closure rate increases linearly with *k*^0^_close_ (a material property) and exponentially with the free energy barrier to closure Δ*G*_close_ (which depends both on material properties such as the bending modulus and the geometry of the tubule). On the other hand, the probability of such a crack annealing decreases with *E*_B_. These observations are consistent with the general principles established from other self-assembly reactions and crystallization (*e.g.*^40,42,49^). When growth rates are sufficiently fast that monomers that associate with strained interactions cannot anneal before additional subunits assemble, defects become locked into the growing structure.

While some potential mechanisms of defect formation are disallowed by the simplifications of our model and simulations, these mechanisms can be neglected in the DNA origami experiments that motivate our work. In particular, the algorithm does not allow for binding between multiple partially assembled structures, but these events are negligible under the dilute assembly conditions with a substantial nucleation barrier that tend to lead to productive assembly.^40,42,50^ Similarly, we do not consider binding of subunits along non-complementary edges because in the experiments,^9^ monomer–monomer interactions were made highly specific using shape-complementary interactions based on blunt-end DNA base stacking, and there is no evidence of significant binding between non-complementary edges in the experiments. Note that it would be straightforward to extend the model to eliminate these simplifications to describe other systems for which these mechanisms are not negligible.

We also note that a kinetic Monte Carlo algorithm can only be reliably mapped to real dynamics if the move set accounts for all relevant transitions that occur in a given system, with approximately correct relative rates for each move. In this respect it is encouraging that the simulated tubule geometry distribution compares well with experimental observations. However, further comparison against additional data will be required to stringently test the simulated dynamics, and to refine relative rates. In particular, the simulations described here suggest that the rate at which free edges within an assembled tubule bind to each other is an important parameter controlling the closure rate and defect formation.

With the availability of additional experimental data, some of these unknown coarse-grained parameters could be directly estimated from experiments. At the same time, these measurements would provide estimates of unknown experimental parameter values. For example, by optimizing simulation tubule geometry and width distributions against experiments performed at different parameter values (*e.g.* target geometry, and monomer concentration), we could estimate the bending rigidity, as well as the closure and growth rates. Additional experimental techniques could enable directly estimating some coarse-grained parameters in the model. For example, growth rates could be estimated from dynamic light scattering experiments of tubule assembly, while dimerization rates and free energies for specific monomer–monomer edge interactions could be estimated from static light scattering experiments of subunits which each has only a single edge activated for binding.^8,9,51^ Angular fluctuations of dimers measured using atomic force microscopy (AFM)^52,53^ or estimated from electron density in cryo-electron microscopy experiments^8,9,54^ would provide an independent means of estimating the bending rigidity.

Through combination with such experimental techniques, our computational and theoretical study could be used to improve the design of existing experimental platforms for tubule assembly. Further, analysis of simulation trajectories for a validated model will provide insights into mechanisms underlying assembly of tubules in these systems, and potentially other related systems with helical geometries such as microtubules,^21–26,55–57^ filamentous viruses,^58–63^ and diverse other helical assemblies found in biological systems.^64^ The model and computational algorithms described in this work are broadly generalizable. They can be readily adapted to other monomer shapes, such as rectangular DNA tiles,^14,48^ wedge-shaped monomers,^23^ or biomolecules such as the tubulin dimers that form microtubules,^21–26,55–57^ and other assembly symmetries, such as icosahedral capsids^31–35,37^ and geometrically frustrated structures.^36^ Such modifications require changing the graph structure of the triangulated sheet and the relationships among the monomer size, monomer–monomer interaction angles, and assembly curvature; for example, ref.37 derived interactions for a model of protein dimer subunits from atomistic simulations of HBV capsids. Thus, this modeling framework can be used to provide similar insights into other assembly geometries with different symmetries and mechanisms of self-limitation.

## Conflicts of interest

There are no conflicts of interest to declare.

## Supplementary Material

SM-018-D2SM00679K-s001

SM-018-D2SM00679K-s002

SM-018-D2SM00679K-s003

SM-018-D2SM00679K-s004
