# Supplementary material for: Polymorphic self-assembly of helical tubules is kinetically controlled
Source: Soft Matter. 2022 Aug 12;18(35):6716–28. doi: 10.1039/d2sm00679k (PMC9472595; doi:10.1039/d2sm00679k)
Supplement: SM-018-D2SM00679K-s004 [file SM-018-D2SM00679K-s004.pdf]

Electronic Supplementary Material (ESI) for Soft Matter.
This journal is © The Royal Society of Chemistry 2022

Supplementary Information for: Polymorphic self-assembly of helical tubules is kinetically controlled

Huang Fang, Botond Tyukodi, Ben Rogers, and Michael Hagan

I. COMPUTATIONAL MODEL AND MONTE CARLO SIMULATIONS

A. Monomer model and tubule lattice structure

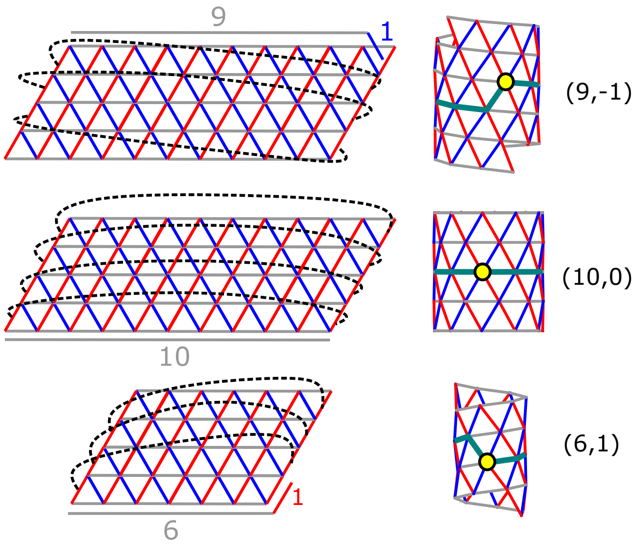

Figure 1. Schematic illustration of the tubule geometry and naming convention. The tubule is represented as a triangulated two-dimensional sheet that is rolled up and closed upon itself. Dashed lines are guides to the eye showing how edges of the rolled-up sheet match and close. A pair of integer numbers (m, n) uniquely identifies each tubule geometry, where m and n respectively correspond to the number of bonds along the circumferential and longitudinal directions along the shortest pathway that travels around the circumference and goes back to the starting vertex. Examples of such pathways are labeled by green lines in the figure, with the starting point labeled by a yellow circle in each case.

Motivated by recent DNA origami experiments in which triangular monomers assembled into icosahedral capsids [1] or helical tubules [2], we consider a model in which triangular monomers assemble into tubules. The tubule structure can be represented as a two-dimensional triangular lattice that is rolled up and closed upon itself in the three-dimensional space. In Fig. §1, we triangulate a two-dimensional sheet and label the three lattice directions in three different colors. Depending on how the edges where the structure closes match, different tubule structures are obtained. We categorize structures using the naming convention employed for carbon nanotubes, in which a pair of integer numbers (m, n) designates a structure [3]. These integers can be identified by starting from an arbitrary vertex, and determining the number of bonds along two directions for the shortest pathway (labeled in green in Fig. §1) that travels around the tubule circumference and returns to the starting vertex. m is the number of bonds with primary orientation in the circumferential direction (gray bonds in Fig. §1) while $|n|$ is the number primarily longitudinal bonds (red or blue bonds). In our convention n is positive for a right-handed helix (if the pathway includes red bonds in Fig. §1) and negative for a left-handed helix (if the pathway includes blue bonds).

B. Calculating preferred dihedral angles for different tubule geometries

To simplify the model, we assume that all triangular monomers are flat, equilateral, and identical. Unlike a flat triangular lattice, the dihedral angles between a triangular monomer and its neighbors cannot all be 180° because the tubule has a non-zero principal curvature along the circumferential direction. To find the preferred dihedral angles, we first calculate the position of vertices. In a cylindrical coordinate system with the origin sitting at the longitudinal axis of the tubule, the radial distances ρ are the same for all the vertices, while the azimuth angles φ and heights z vary. As an example, Fig. §2 shows a schematic of a (6,-2) tubule geometry. We label the diameter of the tubule $D \equiv 2\rho$, the pitch height h of the spiral line formed by gray bonds, the length of edge l_0, the azimuth angle difference $\Delta\varphi_i$, and height difference Δz_i between neighbouring vertices connected by different bonds. $i = 1, 2, 3$ corresponds to blue, red and gray bonds. $\Delta\varphi_i$ and Δz_i are the same for any lattice point because of translation invariance. h is positive for the right-handed tubule and negative for left-handed tubule.

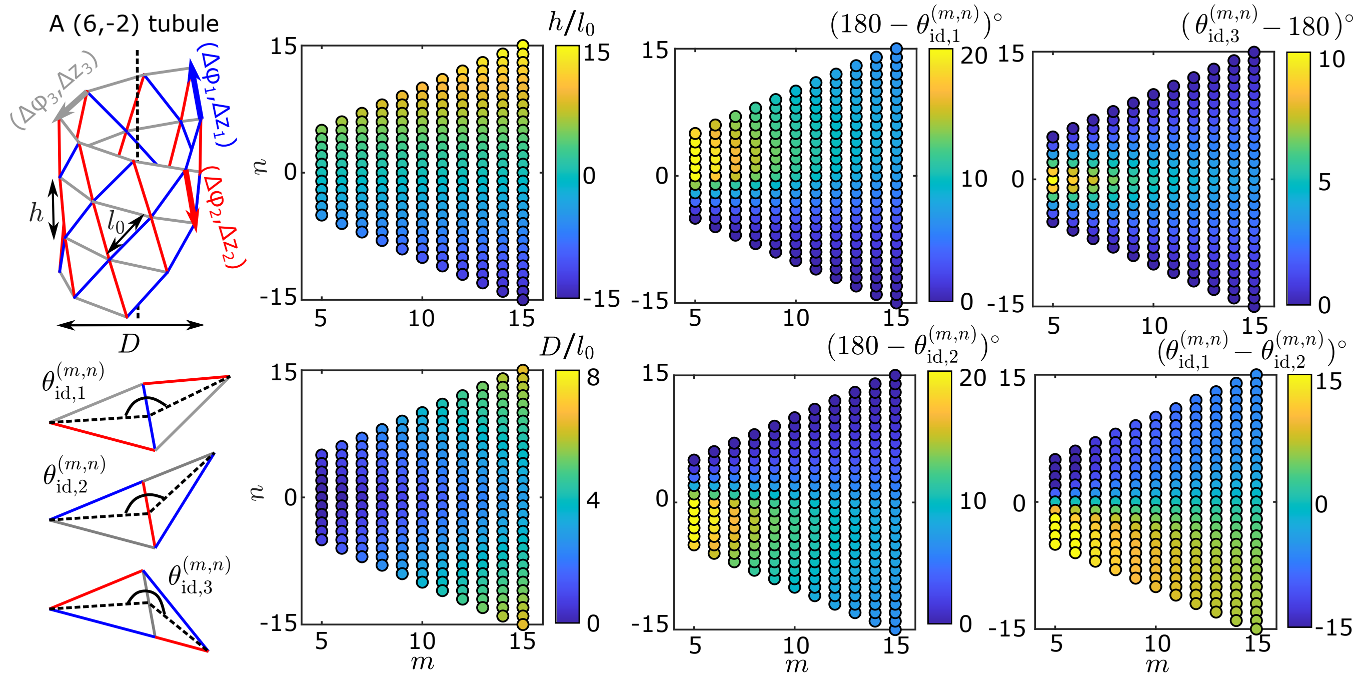

Figure 2. Calculating the geometrical constants for given a tubule geometry (m, n) . The schematic shows a (6,-2) tubule geometry. The diameter D, pitch height h, edge length l_0, and the differences in azimuth angle φ_i and height z_i between neighbouring vertices are labeled. Ideal dihedral angles $\theta_{\rm id,1}^{(m,n)}$, $\theta_{\rm id,2}^{(m,n)}$, and $\theta_{\rm id,3}^{(m,n)}$ correspond respectively to the angles between two adjacent triangular monomers that share a blue, red, or gray bond. The color maps show h/l_0, D/l_0, $180^\circ - \theta_{\rm id,1}^{(m,n)}$, $180^\circ - \theta_{\rm id,2}^{(m,n)}$, $\theta_{\rm id,3}^{(m,n)} - 180^\circ$, and $\theta_{\rm id,1}^{(m,n)} - \theta_{\rm id,2}^{(m,n)}$ for different tubule geometries (m, n).

$\Delta\varphi_i$ and Δz_i of an arbitrary tubule geometry (m, n) should satisfy the following equations,

$$
\begin{gathered}
m\Delta\varphi_3 + \max{(n,0)}\Delta\varphi_1 - \min{(n,0)}\Delta\varphi_2 = 2\pi \\
m\Delta z_3 + \max{(n,0)}\Delta z_1 - \min{(n,0)}\Delta z_2 = 0 \\
\Delta\varphi_1 + \Delta\varphi_2 = \Delta\varphi_3 \\
\Delta z_1 + \Delta z_2 = \Delta z_3 \\
4\rho^2 \sin^2{(\frac{\Delta\varphi_i}{2})} + \Delta z_i^2 = l_0^2
\end{gathered}
$$

We obtain the first two equations by following the shortest pathway that travels around the circumference of the tubule, the third and the forth equation by circulating around the vertices of a triangular monomer, and the final equation based on the equilateral assumption. With these equations, we solve the positions of the vertices given an arbitrary tubule geometry (m, n) as well as the dihedral angles between adjacent monomers. We define ideal dihedral angles $\theta_{\rm id,1}^{(m,n)}$, $\theta_{\rm id,2}^{(m,n)}$ and $\theta_{\rm id,3}^{(m,n)}$ respectively as the angles between adjacent monomers that share blue, red and gray bonds in the tubule geometry (m, n). Fig. §2 shows color maps of D/l_0, h/l_0, $180^\circ - \theta_{\rm id,1}^{(m,n)}$, $180^\circ - \theta_{\rm id,2}^{(m,n)}$, $\theta_{\rm id,3}^{(m,n)} - 180^\circ$, and $\theta_{\rm id,1}^{(m,n)} - \theta_{\rm id,2}^{(m,n)}$ with respect to the tubule geometry numbers (m, n). As m increases, the diameter of the tubule geometry D increases while the pitch height h does not change; both $\theta_{\rm id,3}^{(m,n)}$ and $|\theta_{\rm id,1}^{(m,n)} - \theta_{\rm id,2}^{(m,n)}|$ decrease. All dihedral angles become closer to 180 degrees, since as the diameter of the tubule structure increases, the cylindrical surface becomes closer to a flat surface. As $|n|$ increases, both D and h increase, $\theta_{\rm id,3}^{(m,n)}$ decreases, and $|\theta_{\rm id,1}^{(m,n)} - \theta_{\rm id,2}^{(m,n)}|$ increases. The contributions of $\theta_{\rm id,1}^{(m,n)}$ and $\theta_{\rm id,2}^{(m,n)}$ to the curvature become more asymmetric.

II. COMPUTING TUBULE WIDTH FLUCTUATIONS IN THE CONTINUUM LIMIT

We employ a continuum equilibrium model to compute the width distribution of the assembled tubule structures. Consider a tubule with diameter D and length L. The number of monomers N in the tubule is then given by

$$N = \pi D L / a_0, \tag{1}$$

with a_0 the area of the monomer. Using the Helfrich model for the bending elastic energy, the free energy per monomer is given by

$$g^D = \frac{2a_0\gamma}{L} - \left(\frac{3E_{\rm B}}{2} + Ts\right) + 2\tilde{B}a_0\left(\frac{1}{D} - \frac{1}{D_0}\right)^2 \tag{2}$$

in which $E_{\rm B}$ is the binding energy, γ is the line tension, T is the temperature, and s is the per-monomer entropy in the tubule structure. The coefficient 3/2 in the binding energy term comes from the fact that a triangular monomer has three edges and each bond is shared by two edges. $\tilde{B}$ is the bending modulus (the continuum limit of the discrete bending modulus B) given by [4]

$$\tilde{B} = \sqrt{3}B/2. \tag{3}$$

The equilibrium probability density $\rho^{D,L}$ to assemble a tubule structure with diameter D and length L is given by

$$\rho^{D,L} \cong \exp\left(-\beta N(g^D - \mu)\right) \tag{4}$$

where μ is the chemical potential and N is related to L and D by Eq. (1).

Using this distribution, the root-mean-squared diameter fluctuation ΔD is given by,

$$\Delta D = \sqrt{\frac{\int_0^\infty (D - \langle D\rangle)^2 \exp[-\beta N(g^D - \mu)]dD}{\int_0^\infty \exp[-\beta N(g^D - \mu)]dD}} \tag{5}$$

in which the mean of the distribution $\langle D\rangle$ is given by,

$$\langle D\rangle = \frac{\int_0^\infty D\exp[-\beta N(g^D - \mu)]dD}{\int_0^\infty \exp[-\beta N(g^D - \mu)]dD} \tag{6}$$

The argument of the exponential can be expanded to

$$\begin{aligned}\beta N(g^D - \mu) = \frac{\beta\pi D L(g^D - \mu)}{a_0} &= \beta\pi\left[\left(2\gamma - \frac{L}{a_0}\left(\frac{3E_{\rm B}}{2} + Ts + \mu\right) + \frac{2\tilde{B}L}{D_0^2}\right)D + \frac{2\tilde{B}L}{D} - \frac{4\tilde{B}L}{D_0}\right]\\ &= 2\beta\pi\tilde{B}L\left(\frac{4\gamma a_0 D_0^2 - LD_0^2(3E_{\rm B} + 2Ts + 2\mu) + 4\tilde{B}La_0}{4\tilde{B}La_0D_0^2}D + \frac{1}{D} - \frac{2}{D_0^2}\right)\\ &\equiv 2\beta\pi\tilde{B}L\left(\frac{1}{D^{*2}}D + \frac{1}{D} - \frac{2}{D_0}\right)\end{aligned} \tag{7}$$

$$D^* \equiv \sqrt{\frac{4\tilde{B}La_0D_0^2}{4\gamma a_0D_0^2 - LD_0^2(3E_{\rm B} + 2Ts + 2\mu) + 4\tilde{B}La_0}} = D_0\left[1 + \left(\frac{\gamma}{L} - \frac{3E_{\rm B} + 2Ts + 2\mu}{4a_0}\right)\frac{D_0^2}{\tilde{B}}\right]^{-1/2} \tag{8}$$

To simplify the results, we assume that $\frac{\gamma}{L}$ is negligible in the large L limit. Furthermore, at equilibrium the free energy per monomer of the geometry that minimizes the free energy (in this case the target geometry) is approximately equal to the chemical potential μ [5]. Since the bending energy of the target geometry is zero, the equilibrium chemical potential is approximately $-(\frac{3E_{\rm B}}{2} + Ts^*)$ with s^* the entropy per monomer of the target structure. Assuming the entropy is roughly independent of geometry, we have

$$\left(\frac{\gamma}{L} - \frac{3E_{\rm B} + 2Ts + 2\mu}{4a_0}\right)\frac{D_0^2}{\tilde{B}} \cong 0 \Rightarrow D^* \cong D_0 \tag{9}$$

Then, we can perform a saddle point approximation around $D = D_0$, setting

$$2\beta\pi\tilde{B}L\left(\frac{1}{D^{*2}}D + \frac{1}{D} - \frac{2}{D_0}\right) \cong \frac{2\beta\pi\tilde{B}L}{D_0^3}(D - D_0)^2. \tag{10}$$

This results in the mean diameter $\langle D\rangle = D_0$. Using Eq. (3), ΔD is then given by

$$\Delta D = \sqrt{\frac{\sqrt{3}D_0^3 k_{\mathrm{B}}T}{6\pi BL}}. \tag{11}$$

III. ANALYZING THE DYNAMICAL SIMULATIONS

A. Identification of the tubule geometry

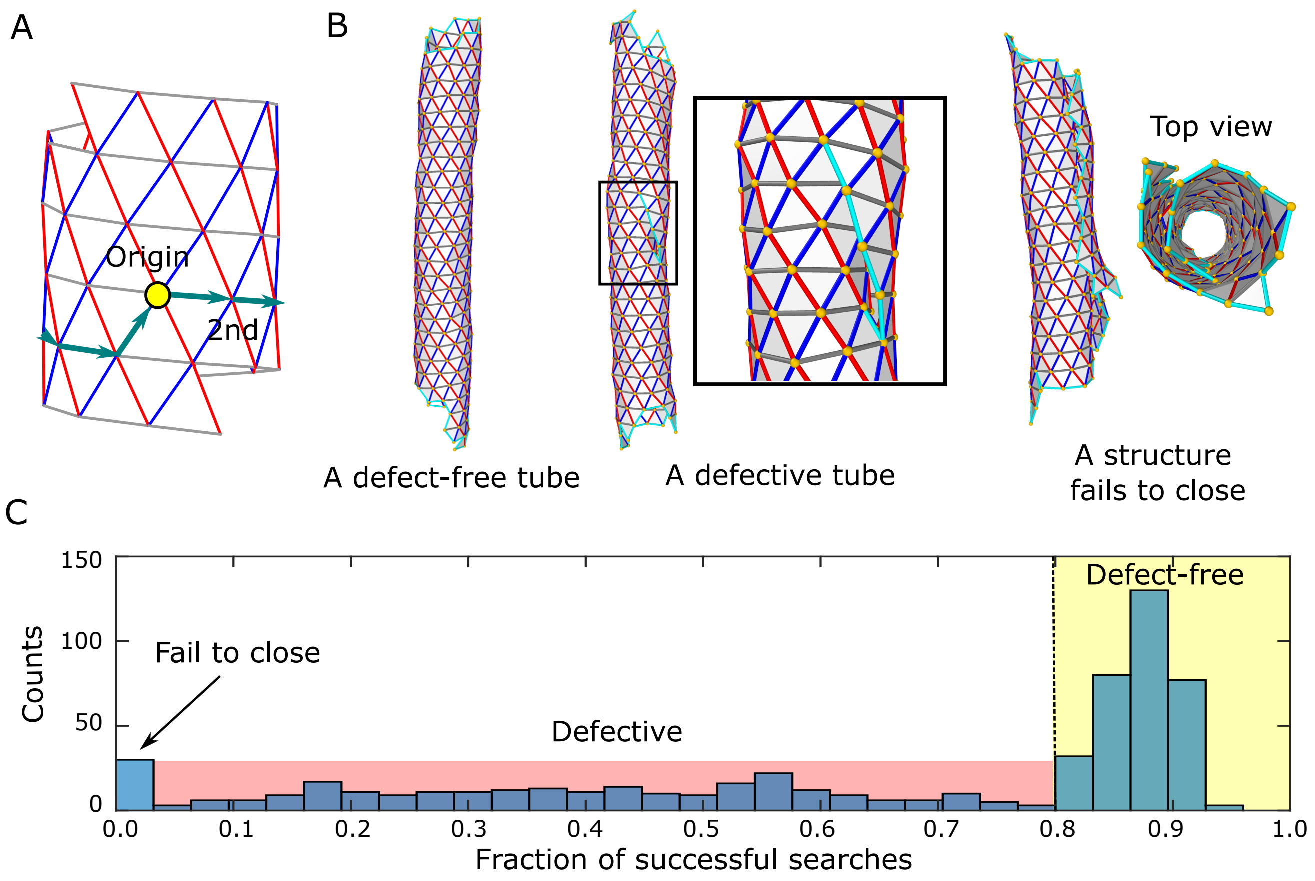

Figure 3. The algorithm for identifying tubule types and determining their distributions. (A) illustrates our search algorithm for determining the tubule type. The yellow circle shows the origin, while the solid turquoise arrows show the shortest pathway. (B) shows examples of defect-free, defective tubules and structures that fail to close. In the snapshots, boundary edges are labeled in cyan. (C) shows the distribution of the fraction of successful searches for 500 simulations run with the same set of parameters. We perform the search algorithm for all the vertices in the final structure and compute the fraction of successful searches for each simulation. We identify a structure as a defect-free tubule only when over 80 % of the searches are successful. Simulation parameters: $E_{\mathrm{B}} = 6.4\ k_{\mathrm{B}}T$, $B = 25\ k_{\mathrm{B}}T$, the target tubule geometry is (15,0). f_{insert} is the attempt frequency of insert/delete move. $f_{\mathrm{insert}} = 0.001$ for all the figures except for Fig. §14.

We classify the structures that form in the simulations by searching for the shortest path around the circumference of the structure, moving along the edges of the triangles. Fig. §3(A) illustrates one such path. We choose a random vertex as the origin. Then, we search for the next vertex connected to the origin by a gray edge, which we call the

second vertex. The vector pointing from the origin to the second vertex defines the direction of the search. Then, we search for vertices along either the red or the blue edges and measure the distance between those vertices and the origin. If those vertices are farther from the origin, we move to the next vertex that is connected through a gray edge. If those vertices are closer to the origin, we move along the red/blue edge until we reach the boundary or return to the origin. If the search along red or blue edges reaches the boundary, we move to the next vertex that is connected through a gray edge. This search continues until we return to the origin or terminates if the last vertex connected through a gray edge is on the boundary. If the search can return to the origin, we label it as a success, otherwise we label it as a failure. The lattice numbers m and n are determined by the number of edges traversed on this pathway. m is the number of the gray edges, while $|n|$ is the number of red or blue edges. n is negative if the pathway follows blue edges (a left-hand helix) and positive if the pathway follows red edges (right-hand helix).

Fig. §3(B) shows examples of defect-free tubules, defective tubules, and structures that fail to close. To identify defective tubules and to prevent incorrect identification, we perform the search algorithm described above for every vertex in the final assembly, and then record the successful search rate (i.e., the fraction of starting vertices for which the search algorithm is successful). Fig. §3(C) shows a distribution of the fraction of successful searches, measured for 500 independent simulations under the same parameter set. By comparing the simulation snapshots and the distribution, we find that the tubes are mostly defect-free around the peak ($\approx$ 0.9 success fraction) and mostly defective when the fraction of successful searches is below 0.8 or multiple tubule geometries are identified in the same structure. That is, we identify a structure as defect-free only if more than 80% of the searches find the same tubule geometry.

B. Diameter measurement

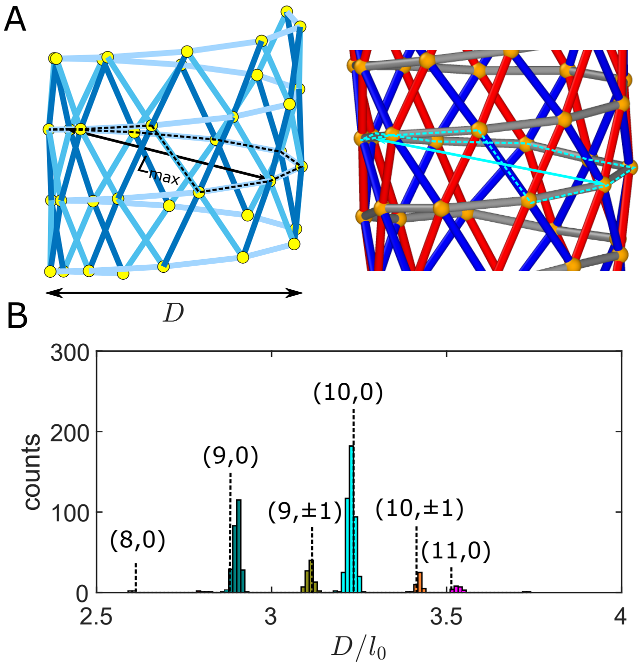

Figure 4. Measuring the diameters of tubules assembled in simulations. (A) Illustration of the measurement algorithm. The left panel shows the structure of a stress-free tubule. $L_{\max}$ (solid line) is the distance between two vertices that are farthest apart within the same *lap*, which is the shortest pathway that traverses the circumference of the structure and returns to the original vertex (dashed line). D is the diameter of the stress-free tubule. R_{LD} is defined as the ratio between $L_{\max}$ and D for calibration. To get the diameter of an assembled tubule, we pair vertices that are the farthest apart within the same lap and measure the distance between those pairs. We compute $\langle L_{\max}\rangle_{\text{sim}}$ by averaging over all paired vertices within the same lap. The local diameter D_{local} of the assembled tubule in the simulation is computed by $\langle L_{\max}\rangle_{\text{sim}}/R_{\text{LD}}$. We compute the diameter of the tubule as the mean value of D_{local} computed from all laps within the structure. (B) A detailed diameter distribution of 500 defect-free tubule structures assembled under the same parameters set. The colors of the bars represent different tubule geometries. Vertical dashed lines label the diameters of the ideal tubule geometries. Simulation parameters: $E_{\text{B}} = 6.0\ k_{\text{B}}T, B = 25\ k_{\text{B}}T$, the target tubule geometry is (10,0), and $f_{\text{fusion}} = 0.01$.

Fig. §4(A) shows how we measure the diameter of the tubule structure we obtained from the simulation. Basically, we perform this measurement by comparing the tubule assembled in the simulation with the stress-free tubule as solved in the previous section I B. The diameter of the stress-free tubule is well defined because all the vertices are on the same cylindrical surface. The diameter of the tubule D is defined as the diameter of the cylindrical surface where vertices sit. On the other hand, we identify the shortest pathway that traverses the circumference of the tubule and returns to the original vertex (the dashed black line on the right panel in Fig. §4(A)), which we call a *lap*. Within the lap, we find vertices that are the furthest apart and define the distance between them as $L_{\max}$ (labeled by the solid black line). We compute $R_{\rm LD}$ as the ratio between $L_{\max}$ and D. $R_{\rm LD}$ is different for different tubule geometries. It increases as the tubule becomes more chiral. We use $R_{\rm LD}$ to calibrate the local diameter of the tubule assembled in the simulation.

To get the diameter of a tubule assembled in the simulation, we first identify all the laps within the tubule structure. Then within each lap, we pair vertices that are the farthest apart and compute $\langle L_{\max}\rangle_{\rm sim}$ by averaging over all paired vertices. The local diameter $D_{\rm local}$ of the lap is computed by $\langle L_{\max}\rangle_{\rm sim}/R_{\rm LD}$. Finally, we compute the diameter D of the structure as the mean value of $D_{\rm local}$ computed from all laps within the structure. Fig. §4(B) shows a detailed distribution of the measured tubule diameter values D. The counts are labelled by color based on the tubule geometry. We can see that fluctuations of D within the same tubule geometry are small, and the mode of each distribution is close to the diameter of the ideal tubule geometry (labeled in dashed lines). The fluctuations around each peak come from thermal fluctuations of each particular tubule geometry itself. The observed fluctuations of tubule width are thus primarily determined by assembly of different tubule structures, not by our measurement method.

C. Closure size measurement

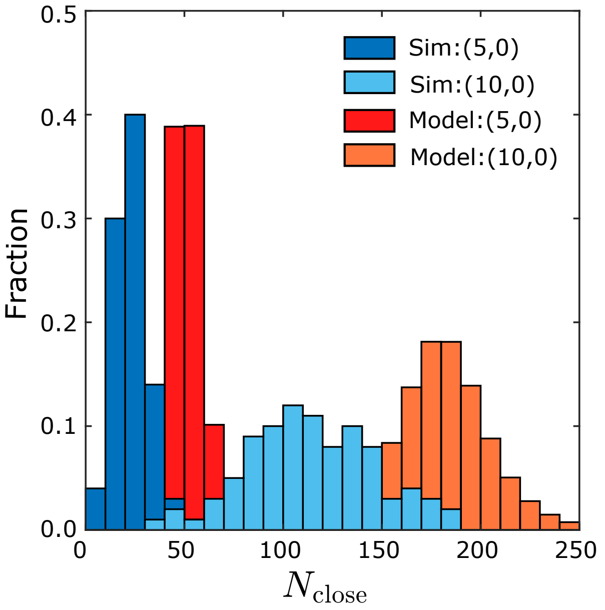

Figure 5. Distribution of closure sizes $N_{\rm close}$ for different target tubule geometries in simulations and the discrete kinetic model (Eq.(12) in the main text). Dark and light blue bars represent the distributions measured from the simulations for the indicated target geometry, while red and orange bars represent the theoretical predictions. Parameter values are: $E_{\rm B} = 5.6\ k_{\rm B}T, B = 25\ k_{\rm B}T, f_{\rm fusion} = 0.001$.

In dynamical assembly trajectories, we define the *closure size* $N_{\rm close}$ as the at structure size N at which an edge fusion move is accepted for the first time in that trajectory. Although it is possible for the fused edge to reopen, the probability of reopening is low and the error this contributes to the closure size measurement is small. Fig. §5 shows closure size distributions for different target tubule geometries measured from simulations, compared with the kinetic model prediction (Section III D of the main text). The mean closure sizes $\langle N_{\rm close}\rangle$ for the target geometries (5,0) and (10,0) are respectively $\langle N_{\rm close}\rangle = 22$ and 120. Note that, in addition to target geometry, the closure size distribution is affected by the binding energy, the bending modulus, and the effective closure frequency $f_{\rm fusion}$. However, within the parameter ranges discussed in the main text, the variations in $\langle N_{\rm close}\rangle$ with different values of these parameters

for a given target geometry are typically small ($\lesssim 20\%$).

The length of a tubule at the point of closure L_{close} can be approximately related to the closure size by

$$L_{\text{close}} \pi D_0 = \langle N_{\text{close}} \rangle a_0 \tag{12}$$

in which D_0 is the diameter of the target geometry and a_0 is the area of the monomer. By comparing L_{close} and D_0 at different target geometries, we find L_{close} typically varies in the range $1.3D_0 \lesssim L_{\text{close}} \lesssim 1.7D_0$. We use $1.5D_0$ in the main text for estimating the equilibrium tubule geometry distribution at the closure size.

We find that the closure size predicted by the kinetic model is approximately twice as large as the simulation result (Fig. §5). We hypothesise this is due to the difference between the preclosure geometry we assume in the model and the actual geometry of the structure assembled in the simulation. In the model, we assume that the open structure has a circular shape with a smooth boundary. However, the structure in the simulation has a coarser and more irregular boundary due to the triangular lattice geometry, entropic edge fluctuations, and the nonequilibrium nature of assembly trajectories. The boundary roughness and non-circular shape may favor closure of the structure at a smaller sizes, by bringing distal edges into closer proximity for a given bending energy cost than would be possible for a perfectly circular structure. Nevertheless, despite this discrepancy the kinetic model reproduces the probability distribution measured from the simulation with reasonable accuracy.

D. Closure stabilizes the tubule geometry

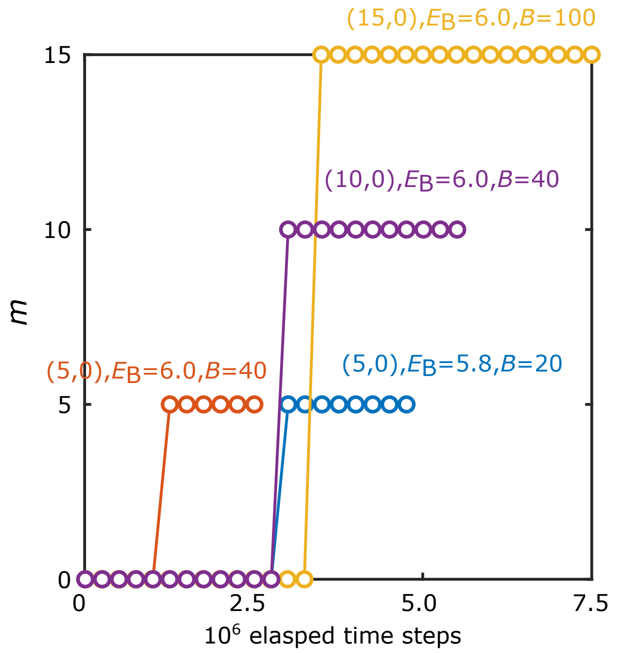

Figure 6. The tubule geometry becomes independent of time after closure. The figure shows the measured tubule geometry as a function of time for four example trajectories at indicated values of the binding energy, bending modulus, and target tubule geometry. In all these examples, the assembled tubules are achiral and they have $n = 0$, and we set $m = 0$ when the structures are open. The parameter $f_{\text{fusion}} = 0.001$ for all the examples.

Performing the tubule-geometry identification at different time points along simulation trajectories shows that the tubule geometry is essentially fixed after closure. Fig. §6 shows identified tubule geometries as a function of time for a selection of trajectories at different parameter sets. In all cases, once closure occurs, the tubule geometry is fixed for the remainder of the simulation. This observation is consistent over the full range of parameters that we explore.

E. Tubule closure probability

Fig. §7 compares the simulation results and kinetic model predictions for the fraction of trajectories that resulted in closed tubules. Structures that failed to close formed paper roll structures (Fig. §3(B)). Results are shown for indicated values of the effective closure rate f_{fusion}, which we controlled by varying the frequency of edge fusion/fission attempts. As f_{fusion} increases by two orders of magnitude, the closure probability increases from 55 % to $\sim$ 100 %. The kinetic model predictions capture this trend and nearly quantitatively agree with the simulation results.

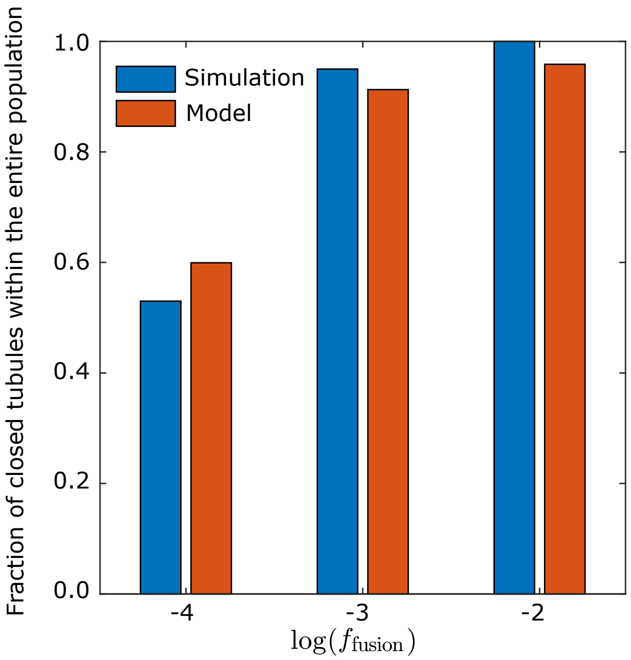

Figure 7. Fraction of simulation trajectories resulting in closed tubules compared against the kinetic model prediction, for $E_{\text{B}} = 6.0\ k_{\text{B}}T, B = 20\ k_{\text{B}}T$, and the target tubule geometry (10,0).

F. Tubule growth rates

To describe the growth dynamics, we define the growth rate k_{grow} as the steady-state time-derivative of the structure size N. Fig. §8(A) shows how we measure k_{grow}. We see that once the tubule structure has closed and filled gaps in the structure to achieve a roughly steady-state morphology, the net growth rate fluctuates around a constant value. The fluctuations arise due to stochastic association events and because monomers can detach from the structure (in particular from the two open ends where monomers have fewer contacts) due to the relatively weak binding energy E_{B} required for assembly of well-formed tubules. To estimate the net growth rate, we choose a time point τ_0 and perform a local time-average by performing linear regression on N as a function of time τ within a range of 2×10^5 time steps centered at τ_0, and compute the slope of the linear fit. Designating the structure size at τ_0 as N_0, the slope of this linear fit provides one measurement of the growth rate at size N_0. We compute the growth rate by averaging over 500 measurements under the same parameter for each value of N or N_{boundary}. We select 2×10^5 time steps as the fitting range because this is typically the smallest time interval for which there is a significant change in N (> 10) beyond the critical nucleus size N_{C} within the parameter space that we simulated. By choosing the smallest such time interval, we avoid over-smoothing fine features on the N, τ curve.

Fig. §8(B) shows k_{grow} as a function of N for different assembled tubule geometries (colored symbols), as well as points along the trajectories before tubules have closed (open symbols). We observe that k_{grow} increases with N before closure and plateaus after closure once the tubule geometry reaches a steady state. The steady-state growth rate increases with the width of the assembled tubule geometry. The inset of Fig. §8(C) shows that the growth rate of closed tubules is proportional to m, which determines the tubule diameter and thus the mean value of N_{boundary} for a closed structure.

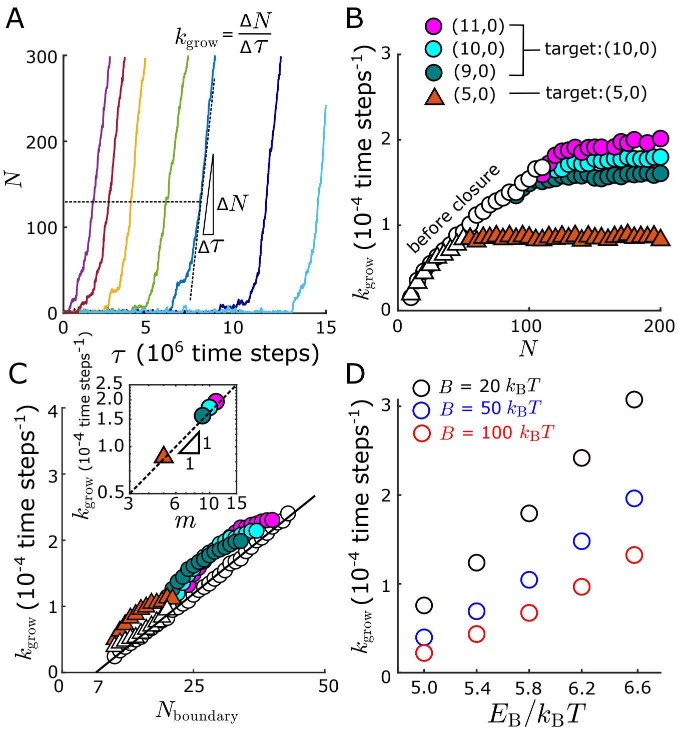

Figure 8. Measuring the growth rate $k_{\rm grow}$ from simulation trajectories. (A) Structure size N as a function of elapsed time step τ (Monte Carlo sweep). The growth rate $k_{\rm grow}$ is defined as the first order derivative of N with respect to τ, which is locally time-averaged as described in the text. (B) Measured growth rates for different assembled tubule geometries (indicated by symbol color) and different target geometries (indicated by symbol type). Points along trajectories before the structure has closed are indicated by open symbols. Each data point is an average over 500 independent trajectories. (C) Growth rate as a function of the number of edges on boundary $N_{\rm boundary}$. Symbols have the same meaning as (B). The solid line is a linear fit to the data corresponding to the white circles. In the inset, m is the first lattice number of the assembled tubule geometry (m, n), with $n = 0$. The dashed line is a guideline to eyes for a slope of 1. Each data point is an average over 500 measurements. (D) Growth rate of assembled tubules with geometry (10,0) as a function of binding energy for indicated values of the bending modulus. Simulation parameters in (A),(B), and (C) are: $E_{\rm B} = 5.8\ k_{\rm B}T, B = 20\ k_{\rm B}T$.

To understand these trends, we measured $k_{\rm grow}$ as a function of the number of boundary edges $N_{\rm boundary}$ (Fig. §8(C)). We find that the growth rate increases roughly linearly with $N_{\rm boundary}$, but with a non-zero intercept of $N_{\rm boundary} \approx 7$. The non-zero intercept is reasonable since the growth rate should approach zero as N approaches the critical nucleus size $N_{\rm C}$. For this parameter set, $N_{\rm C} = 5$ monomers, and the number of boundary edges at the critical nucleus size ranges from $7 \lesssim N_{\rm boundary} \lesssim 12$, consistent with the observed intercept. We observe that the steady-state growth rate for closed structures is somewhat larger than that for open structures, but is roughly linearly related to the number of boundary edges $N_{\rm boundary}$. This trend is consistent with stable monomer attachment events at each open edge, as would be expected for conditions under which monomer addition is significantly biased over monomer detachment. However, the net growth rate is somewhat smaller for open structures compared to closed structures at the same value of $N_{\rm boundary}$. This trend can be understood from the fact that addition of a monomer with only a single contact is relatively unstable at these parameter values due to binding entropy penalties. Thus, stable monomer addition requires formation of at least two contacts. Formation of two (or more) contacts occurs more readily for a closed structure compared to an open structure due to the larger curvature of the rim in the latter case.

Despite the geometrical differences between open and closed tubule structures, $k_{\rm grow}$ depends linearly on $N_{\rm boundary}$ during growth in both phases. Below closure, considering the limit that the critical nucleus size is small and $N_{\rm boundary}$ scales with $\sqrt{N}$ before closure, $k_{\rm grow}$ can be approximated by

$$k_{\rm grow}(N) = k^0_{\rm grow}\sqrt{N} \tag{13}$$

in which $k^0_{\rm grow}$ is a factor that can be measured from the simulation, which depends on B, $E_{\rm B}$ and the attempt frequency of the monomer insertion/deletion moves. Fig. §8(D) shows the growth rate $k_{\rm grow}$ of assembled tubules with geometry (10,0) measured at structure size $N = 150$ ($k_{\rm grow}$ becomes independent of size shortly after the tubule closes). $k_{\rm grow}$ increases as $E_{\rm B}$ increases and B decreases.

G. Closure

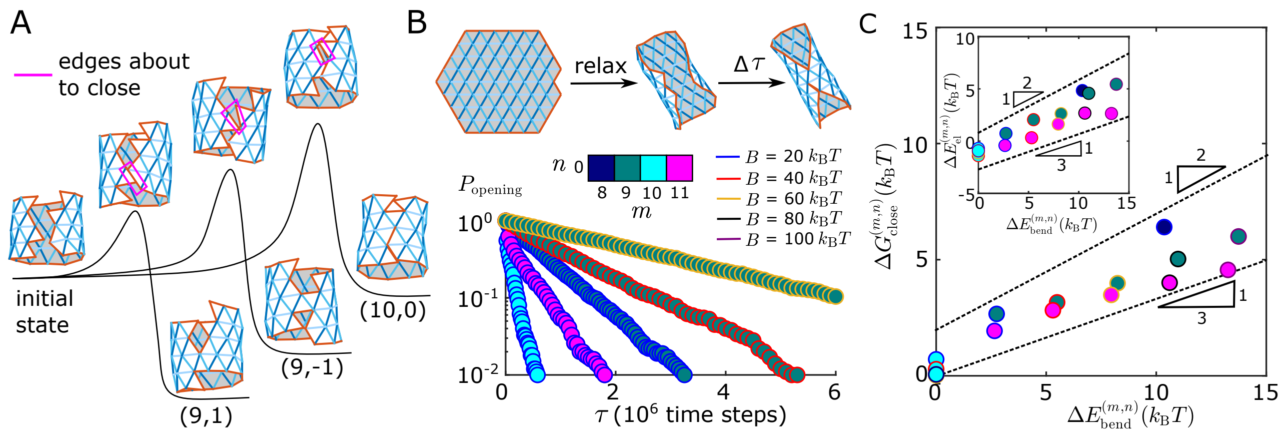

Figure 9. Measurement of the closure rate k_{close} as a function of the bending modulus and closed tubule geometry. **(A)** Schematic of the closure process and associated free energy barrier. The edges labeled in magenta are the edges that are about to close. Starting from a given initial state, the system can close into different tubule geometries, each of which has a different free energy barrier to closure. **(B)** (top) Schematic of the process to measure closure rates from Monte Carlo simulations. We first let the flat sheet relax to an equilibrium distribution by only allowing vertex moves. Then we enable edge fusion/fission moves and vertex moves and measure the faction of structures that remain open as a function of the elapsed time. Different face colors represent different tubule geometries that the structure closes into. Different edge colors represent different values of B. **(C)** Comparison between the free energy barrier and the estimated bending elastic energy difference between open and closed structures. The symbols have the same meaning as in (B). The inset compares the elastic energy difference measured from the simulations and the estimated bending elastic energy difference. Simulation parameters are : $E_{\text{B}} = 6.0\,k_{\text{B}}T, f_{\text{fusion}} = 0.001$, and the target geometry is $(10, 0)$.

Except when the growing disk has a size commensurate with closure of a stress-free tubule (that is, the diameter of the disk is related to the preferred diameter of the tubule by $D_{\text{disk}} = \pi D_0$ so that when the edges of the disk touch each other the structure has the stress-free-curvature), there will be a free energy barrier to closure arising from the bending energy required to adopt a curvature (i.e. monomer-monomer binding angles) that differ from the preferred geometry. In particular, the disk must curve in such a way that its edges approach each other closely enough to bind. Once these contacts are made and the monomers bind, then the additional binding energy can compensate the unfavorable bending energy. Thus, an estimate of the free energy barrier to closure can be obtained by computing the bending energy difference between the disk at the point just before closure occurs, and in its stress-free state. Here, we are making the good approximation that we can neglect configurational entropy differences between these states.

Fig. §9(A) shows a schematic of the closure process. Depending on the size of the disk, different tubule geometries can be formed by closure, and the free energy barrier is a function of both the disk size and the geometry being formed.

We have used several approaches to compute the closure free energy barrier. First, we measured the closure rates for an ensemble of unclosed proto-tubule configurations, constructed to have well-formed triangular lattices such as the ones that occur during assembly dynamics under productive assembly conditions (Fig. §9(B)). The pre-closure structure that we choose resembles a regular hexagonal geometry if flattend onto the two dimensional plane, yet with a 'notch' for accepting the edge fusion move. The reason to choose this spatial configuration is that we have identical binding energies for all three edges and the growth is nearly isotropic before closure. We can use a pair of numbers $[a, b]$ to describe the geometry of the pre-closure structure, in which a is the number of triangles along the long (horizontal) axis and b is the number of triangles along the short (vertical) axis of the flattend pre-closure structure. The pre-closure structures we use for the closure rate measurement are [8,8], [9,8], [10,10] and [11,10], which assembles (8,0), (9,0), (10,0), (11,0) tubules when they close. The aspect ratio of these pre-closure structures is roughly held the same. The target tubule geometry is set to (10,0) for all the closure rate measurements, however, the pre-closure structure limits the tubule geometries that can assemble via closure.

First, we generate each pre-closure structure as a two dimensional triangular lattice using a home-made MATLAB code and use that as the initial state in our dynamical simulations. Then, we perform only vertex moves to equilibrate the configuration at fixed size and topology with the preferred dihedral angles favouring a (10,0) tubule. We performed 10^4 sweeps to make sure that the pre-closure structure equilibrated. Finally, we allowed both vertex and edge fission/fusion moves (so that the structure can close), and measured the fraction of structures that remain open as a

function of elapsed time. We performed this measurement for various diameters (the long axis of the open structure, which equals the circumference of the closed structure) and B values for a target geometry of (10,0).

The bottom plot in Fig. §9(B) shows that the fraction of open structures decays exponentially as a function of time. By performing an exponential fit with decay time τ_c for each data set, we extract the closure rate $k_{\text{close}} = 1/\tau_c$. We observe that k_{close} decreases as the bending modulus B increases or the assembled tubule geometry deviates further from the target tubule geometry.

Because the measured closure time distributions are exponential, we assume that the closure rate follows the Arrhenius mechanism, and we estimate the closure free energy barrier $\Delta G^{(m,n)}_{\text{close}}$ from

$$k_{\text{close}} = k_0 \exp\left(-\Delta G^{(m,n)}_{\text{close}}/k_{\text{B}}T\right)$$

with k_0 as the closure rate when the diameter of the unclosed structure equals the circumference of the target tubule geometry.

Fig §9(C) compares the measured $\Delta G^{(m,n)}_{\text{close}}$ to the estimated bending energy difference $\Delta E^{(m,n)}_{\text{bend}}$. Considering that all monomers in the open structure have dihedral angles that favor the tubule geometry (m, n), the bending elastic energy difference between such any configuration and the stress-free configuration $\Delta E^{(m,n)}_{\text{bend}}$ is given by

$$\Delta E^{(m,n)}_{\text{bend}}(N) = \frac{1}{4} BN \sum_{i=1,2,3} (\theta^{(m,n)}_{\text{id},i} - \theta^0_{\text{id},i})^2. \tag{14}$$

We observe that $\Delta G^{(m,n)}_{\text{close}} \approx \alpha \Delta E^{(m,n)}_{\text{bend}}$ with $0.3 \lesssim \alpha \lesssim 0.5$ as discussed in the main text. Therefore, the closure rate can be approximated by,

$$k^{(m,n)}_{\text{close}}(N) = k^0_{\text{close}} \exp\left(-\alpha \Delta E^{(m,n)}_{\text{bend}}/k_{\text{B}}T\right). \tag{15}$$

In the inset of Fig. §9(C), we also compare the elastic energy difference measured in the simulation $\Delta E^{(m,n)}_{\text{el}}$ to the bending energy estimate $\Delta E^{(m,n)}_{\text{bend}}$. We measured $\Delta E^{(m,n)}_{\text{el}}$ as the difference between the elastic energy just before the structure closed and the average elastic energy of the structure at equilibrium (with fixed size and topology). Notice that we observe the same scaling, $\Delta E^{(m,n)}_{\text{el}} \approx \alpha \Delta E^{(m,n)}_{\text{bend}}$, which supports the assumption above that the closure free energy barrier arises primarily due to the elastic energy difference between the closed and open configurations. Nevertheless, the measured elastic energy difference is only $\sim 30\,\%$ of the elastic energy if the entire unclosed structure adopts a curvature commensurate with the closed structure (inset of Fig. §9(C)). We hypothesize that this discrepancy arises because the bending is nonuniform and only a few contacts are required to initially stabilize the closed structure. For the kinetic model, we use $\alpha = 0.3$ to account for these effects.

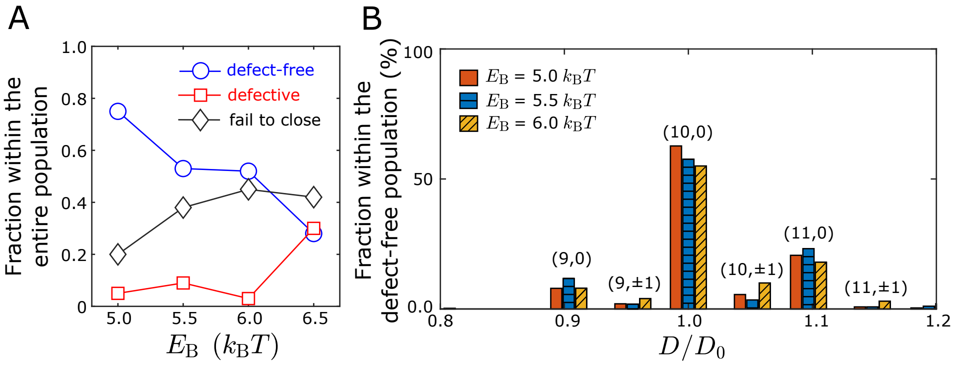

Figure 10. The effect of varying the binding energy E_{B} on tubule assembly geometries, observed in dynamical assembly simulations. **(A)** The fraction of defect-free tubules, defective tubules and unclosed structures within the entire population. The distributions were estimated from 1000 independent simulation trajectories for each value of E_{B}. **(B)** Geometry distributions within the defect-free population, for indicated values of E_{B}. The paired numbers with parentheses indicate the tubule geometry. D_0 is the diameter of the ideal (10,0) tubule. Other simulation parameters are: target geometry (10,0), $B = 20\ k_{\text{B}}T$, $f_{\text{fusion}} = 0.0001$.

H. The dependence of assembled tubule geometries on binding energy

Fig. §10 shows the geometry distributions of tubules assembled in simulations with target structure (10,0) and different values of the binding energy E_{B}. As E_{B} increases, the fraction of defect-free tubules decreases, while the fraction of defective tubules and open structures increases. This is consistent with observations in other self-assembly systems that the probability of defective structures increases with binding energy because the rate of annealing defects decreases exponentially with increasing binding energy [6, 7]. However, the geometry distribution within the defect-free population does not change significantly. This can be understood as follows, based on the results of the kinetic model (section III of the main text). The tubule closure size is substantially larger than the critical nucleus size for the parameter regime that we focus on, so the growth dynamics is strongly forward-biased and thus the growth rate depends only weakly on E_{B} (or μ). Therefore, although the kinetic model predicts that the width distribution will shift to larger values with increasing growth rate, this effect will be very weak with increasing E_{B} when the closure size is large compared to the critical nucleus size.

IV. COMPARING GEOMETRY DISTRIBUTIONS OF TUBULES ASSEMBLED IN SIMULATIONS AND EXPERIMENTS

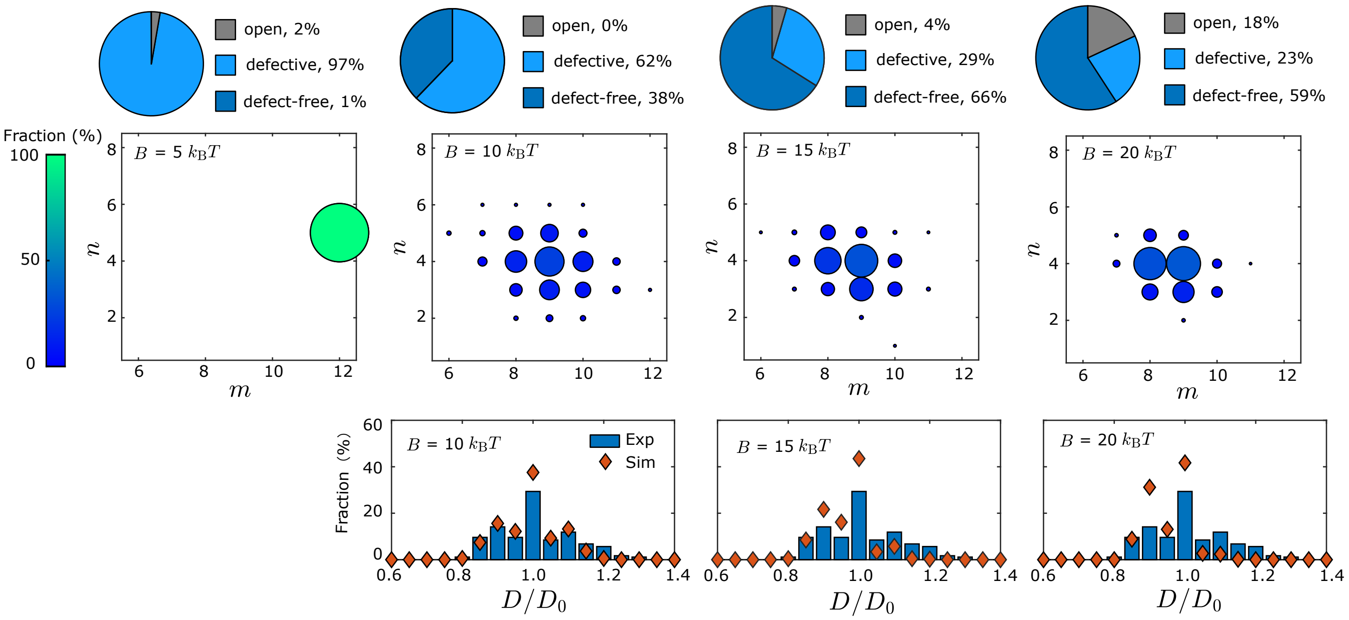

Figure 11. Comparison of geometry distributions for tubules assembled in simulations and experiments. (top) Distribution of geometries of tubules assembled in simulations with indicated values of the bending modulus. The distributions were estimated from 1000 independent simulation trajectories for each value of B. The pie charts show the fractions of defect-free tubules, defective tubules, and open structures. For each B, the size and color of each circle in the middle panel indicates the fraction of the corresponding tubule geometry within the defect-free population. (bottom) Comparison of the tubule diameter distribution between experiments and simulations with different values of the bending modulus. D_0 is the diameter of the ideal (9,4) tubule. Simulation parameters are: the target geometry is (9,4), $E_{\mathrm{B}} = 6.0\ k_{\mathrm{B}}T$, $B = 5, 10, 15, 20\ k_{\mathrm{B}}T$ from left to right, $f_{\mathrm{fusion}} = 0.001$.

Fig. §11 shows the geometry distributions of tubules assembled in simulations with target structure (9,4) and different values of the bending modulus. A high fraction ($\sim 95\,\%$) of the assembled structures are defective for $B = 5\,k_{\mathrm{B}}T$. For $B \geq 10\ k_{\mathrm{B}}T$, the majority of tubules are well-formed, with distributions peaked around the target geometry of (9,4) and the spread comparable to the experimental measurement. As B increases, the distribution of tubule geometries becomes progressively narrower. The experimental distribution in different panels of Fig.§11 is from the same measurement

V. SUPPLEMENTARY INFORMATION ABOUT THE KINETIC MODEL

A. Schematic of the kinetic model

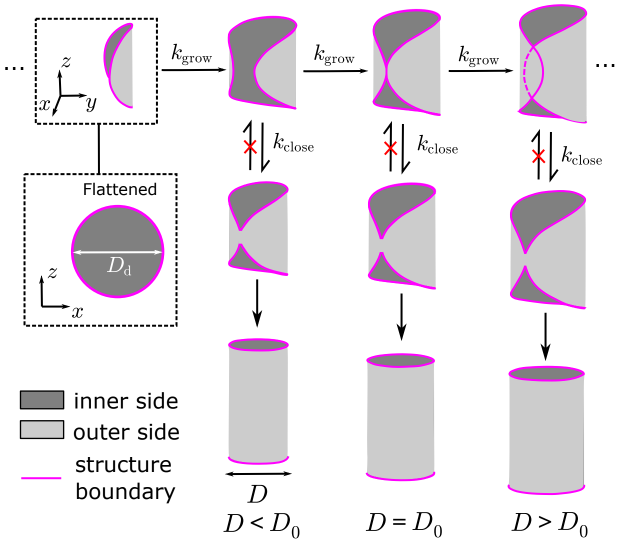

Figure 12. Schematic illustration of the kinetic model.

Fig. §12 shows the schematic of our kinetic model. Prior to closure, the assembling structure is modeled as a curved circular disk. The diameter of the flattened disk is D_{disk}. The pre-closure structure grows with a growth rate k_{grow}, and in the mean time, attempts closure with a closure rate is k_{close}. The transitions that are marked with red 'x' symbols in the diagram are considered low probability, which means that the closed structure does not reopen. D is the diameter of the closed assembled tubule structure, while D_0 is the diameter of the target tubule structure.

B. Determining the compatibility function $I^{(m,n)}_{\text{close}}(N)$

$I^{(m,n)}_{\text{close}}(N)$ is a function that indicates whether a structure of size N is geometrically compatible with closing into a structure (m, n). We compute $I^{(m,n)}_{\text{close}}(N)$ in the following simplified manner. Assuming a circular disk forms a cylinder geometry by bending around a single axis and closing where the two outermost points meet, the curvature κ of the closed structure is

$$\kappa(N) = \frac{2\pi}{D_{\text{disk}}} = \sqrt{\frac{\pi^3}{N a_0}}. \tag{16}$$

However, we must account for the fact that only discrete tubule geometries are allowed. In general, closure to many geometries will require fluctuations from the perfect circular disk that we have assumed thus far. In SI section V C 2 and V D, we perform a calculation in which we explicitly compute the Boltzmann-weighted probability of disk shape fluctuations in the continuum limit. That calculation and analysis of aspect ratios of unclosed disks within simulation trajectories show that the magnitude of shape fluctuations is generally small. Therefore, we simplify the calculation here by neglecting shape fluctuations, but assuming that closure only occurs for geometries that have curvatures close to the circular disk curvature: $\kappa \in [(1-\delta)\kappa(N), (1+\delta)\kappa(N)]$. δ is the cutoff threshold δ, which we determined from the extent of aspect ratio fluctuations of open structures within simulation trajectories (See Fig.§13 and SI section V D for details about the aspect ratio fluctuation measurement. Within the parameter space that we have studied, the surface energy is primarily determined by the binding energy, and the shape distribution of disks near closure size does not vary dramatically with B. Thus, for simplicity we use the same value of δ for all parameters with the same target geometry. We use $\delta = 0.05$ for the $(10, 0)$ target.

The accessibility indicator function is then given by

$$I_{\text{close}}^{(m,n)} = \Theta\left[1 - \frac{1}{\delta}\left|\frac{\kappa^{(m,n)}}{\kappa(N)} - 1\right|\right] \tag{17}$$

with Θ the Heaviside function.

C. Continuum limit

In the main text, we introduce a discrete kinetic model that reproduces the distribution measured in the simulation. Here, we perform an analogous calculation in the continuum limit. We find that the resulting model qualitatively matches the simulation results, but is not as quantitatively accurate as the discrete model.

1. Model without shape fluctuations

First, we assume that the preclosure geometry of the structure is always circular, without shape fluctuations. If such structure closes, the diameter D of the closed tubule is given by

$$Na_0 = \frac{\pi D_{\text{disk}}^2}{4} \tag{18}$$

$$\Rightarrow D_{\text{disk}} = 2\sqrt{\frac{Na_0}{\pi}} \tag{19}$$

where N is the number of monomers in the assembled structure, a_0 is the area of each monomer, and D_{disk} is the diameter of the flattened disk. If the disk closes, D_{disk} will equal the circumference of the closed tubule. Thus, the diameter D of the closed tubule is related to the structure size N by

$$D = \frac{D_{\text{disk}}}{\pi} = 2\sqrt{\frac{Na_0}{\pi^3}} \tag{20}$$

In terms of the kinetic rates, we again approximate k_{grow} by

$$k_{\text{grow}}(N) = k_{\text{grow}}^0\sqrt{N} \tag{21}$$

where k_{grow}^0 is a kinetic factor that can be measured from simulations. The closure rate k_{close} is approximated by

$$k_{\text{close}}(N) = k_{\text{close}}^0 \exp\left(-\alpha\Delta E_{\text{bend}}\right) \tag{22}$$

We use the Helfrich energy model to estimate the bending energy differences between tubules with different diameters [8].

$$\Delta E_{\text{bend}} = 2\tilde{B}Na_0\left(\frac{1}{D} - \frac{1}{D_0}\right)^2 \tag{23}$$

where D is the diameter of the tubule that the open structure is about to close into, D_0 is the diameter of the target tubule geometry, and $\tilde{B}$ is the effective bending modulus at the continuum limit, which is related to the bending modulus B of the discrete triangular lattice by $\tilde{B} = \sqrt{3}B/2$ [4]. The closure rate k_{close} can be further simplified to

$$\begin{aligned} k_{\text{close}}(N) &= k_{\text{close}}^0 \exp\left[2\alpha\tilde{B}Na_0\left(\frac{1}{D} - \frac{1}{D_0}\right)^2\right] \\ &= k_{\text{close}}^0 \exp\left[\sqrt{3}\pi^3\alpha B\left(1 - \sqrt{\frac{N}{N_0}}\right)^2\right] \end{aligned} \tag{24}$$

D_0 and N_0 are related by,

$$D_0 = 2\sqrt{\frac{N_0 a_0}{\pi}} \tag{25}$$

where $k^0_{\rm close}$ is a kinetic factor that can be measured from simulations.

Finally, we evaluate the closure probability as a function of time. In the absence of closure, the structure remains at each size N for a time $\Delta t(N) = 1/k_{\rm grow}(N)$, and the time at which a structure first grows to size N is $t_N = \sum_{i=1}^{N-1} 1/k_{\rm grow}(N)$. The probability that a structure with size N remains open at a time $t_N \leq t < t_{N+1}$ is given by

$$P_{\rm open}(t, N) = P_{\rm open}(t_N, N) \exp\left[-k_{\rm close}(N)(t - t_N)\right]. \tag{26}$$

By summing over the smaller sizes, we can compute the probability that a structure has stayed open until size N as

$$P_{\rm open}(t_N, N) = \prod_{i=1}^{N-1} \exp\left(-\frac{k_{\rm close}(i)}{k_{\rm grow}(i)}\right). \tag{27}$$

The probability for the structure to close at size N is then given by

$$P_{\rm close}(N) = \prod_{i=1}^{N-1} \exp\left(-\frac{k_{\rm close}(i)}{k_{\rm grow}(i)}\right) \left[1 - \exp\left(-\frac{k_{\rm close}(N)}{k_{\rm grow}(N)}\right)\right] \tag{28}$$

Since D and N are related by Eq.20, the width distribution is equivalent to the closure size distribution.

2. Model with shape fluctuations

To incorporate shape fluctuations of the pre-closure structure into the model, we consider the structure before closure as elliptical rather than circular. The aspect ratio ψ is defined as the ratio between the lengths of the major and minor axes. The orientation angle ϕ is defined as the angle between the major axis and the direction of the non-zero principle curvature (the circumferential direction of the tubule once it closes). We assume that ϕ follows a uniform distribution from 0 to π. Assuming the system is quasi-equilibrated before closure, the distribution f of ψ, ϕ should follow Boltzmann distribution. According to classical nucleation theory (CNT), the free energy barrier G to forming an open structure from unassembled monomers can be written as

$$G = \Delta g N + \sigma N_{\rm boundary} \tag{29}$$

where Δg is the per-monomer bulk free energy difference between the structure and the fluid phase, σ is the per-monomer surface free energy, N is the number of monomers in the structure, and $N_{\rm boundary}$ is the number of monomers at the structure boundary, which depends on ψ.

Assuming Δg does not change with ψ, the bulk term depends only on N and the shape distribution is completely determined by the surface energy of the open structure. The distribution f of ψ and ϕ at size N is given by

$$f(N, \psi, \phi) = \frac{1}{\pi Z_\psi} \exp\left(-\frac{\sigma L(N, \psi)}{l_0 k_{\rm B} T}\right) \tag{30}$$

where Z_ψ is the partition function obtained by summing over ψ and ϕ, assuming that the latter follows a uniform distribution, which contributes a factor of $1/\pi$. l_0 is the stress-free length of the edge and $L(N, \psi)$ is the perimeter of the open elliptical geometry, which is given by

$$N a_0 = \pi a b = \frac{\pi a^2}{\psi} \tag{31}$$

$$L^{(N,\psi)} = 4a \int_0^{\frac{\pi}{2}} \sqrt{1 - \left(1 - \frac{1}{\psi^2}\right) \sin^2\theta d\theta} \tag{32}$$

where a_0 is the area of each monomer, a is half the length along the major axis, and b is half the length along the minor axis. The first equation enforces that the area of the structure is conserved for fixed N. The second equation

gives the perimeter of the elliptical geometry. The surface energy density σ can be measured from thermodynamic integration as introduced later in SI section VII.

In terms of the kinetic rates, we again approximate k_{grow} by

$$k_{\mathrm{grow}}(N) = k^0_{\mathrm{grow}}\sqrt{N} \tag{33}$$

where k^0_{grow} is a kinetic factor that can be measured from simulations. The closure rate k_{close} is approximated by

$$k_{\mathrm{close}} = k^0_{\mathrm{close}} \exp\left(-\alpha \Delta E_{\mathrm{bend}}\right) \tag{34}$$

We use the Helfrich energy model to estimate the bending energy differences between tubules with different diameters [8].

$$\Delta E_{\mathrm{bend}} = 2\tilde{B} N a_0 \left(\frac{1}{D} - \frac{1}{D_0}\right)^2 \tag{35}$$

where D_0 is the diameter of the target tubule geometry and $\tilde{B}$ is the effective bending modulus at the continuum limit defined above. D is the diameter of the tubule that the open structure is about to close into, and is given by

$$D^{(N,\psi,\phi)} = \frac{2}{\pi}\sqrt{\frac{N a_0 \psi}{\pi(\cos^2\phi + \psi^2 \sin^2\phi)}} \tag{36}$$

This equation is obtained by assuming that the circumference of the closed tubule geometry is equal to the length of the elliptical geometry along the direction of the non-zero principal curvature.

Finally, we evaluate the closure probability as a function of time. In the absence of closure, the structure remains at each size N for a time $\Delta t(N) = 1/k_{\mathrm{grow}}(N)$, and the time at which a structure first grows to size N is $t_N = \sum_{i=1}^{N-1} 1/k_{\mathrm{grow}}(N)$. The probability that a structure with a certain geometry remains open at a time $t_N \le t < t_{N+1}$ is given by

$$P_{\mathrm{open}}(t, N, \psi, \phi) = P_{\mathrm{open}}(t_N, N, \psi, \phi) \exp\left[-k_{\mathrm{close}}(t - t_N)\right] \tag{37}$$

By summing over ψ and ϕ, we can compute the probability that a structure with size N remains open at a time $t_N \le t < t_{N+1}$ as

$$P^{(t,N)}_{\mathrm{open}} = P^{(t_N,N)}_{\mathrm{open}} \int_{\psi=1}^{\psi=\infty} \int_{\phi=0}^{\phi=\pi} f(N,\psi,\phi) \exp[-k_{\mathrm{close}}(t - t_N)] d\phi d\psi \tag{38}$$

By summing over the smaller sizes, we can compute the probability that a structure has stayed open until size N as

$$P^{(t_N,N)}_{\mathrm{open}} = \prod_{n=1}^{N-1} \int_{\psi=1}^{\psi=\infty} \int_{\phi=0}^{\phi=\pi} f(n,\psi,\phi) \exp\left(-\frac{k_{\mathrm{close}}(n,\psi,\phi)}{k_{\mathrm{grow}}(n)}\right) d\phi d\psi. \tag{39}$$

The probability to assemble a tubule geometry with diameter D is then computed by summing over all sizes n that can close into the diameter D,

$$P_{\mathrm{close}}(D) = \sum_{n=1}^{\infty} P^{(t_n,n)}_{\mathrm{open}} \iint_{S_D} f(n,\psi,\phi) \left[1 - \exp\left(-\frac{k_{\mathrm{close}}(n,\psi,\phi)}{k_{\mathrm{grow}}(n)}\right)\right] d\phi d\psi \tag{40}$$

where S_D is the subset in parameter space of ψ and ϕ that enables the structure to close with diameter D.

D. Aspect ratio distribution

Fig. §13 compares the aspect ratio distribution of open structures measured from simulations and computed from the continuum model that considers shape fluctuations (Eqs. (30)-(32)). The structure size is $N = 120$, which is also the average closure size of the (10,0) tubule. To measure the aspect ratio distribution in simulations, we first performed simulations in which the size was allowed to grow to $N = 120$. Then we changed the preferred dihedral angles to zero and increased the bending rigidity to $B = 400\,k_{\mathrm{B}}T$ so that the structures flattened. Then, we rotated

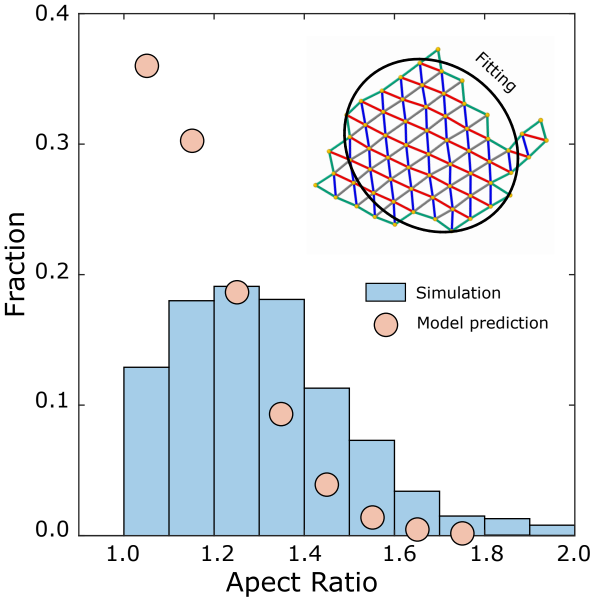

Figure 13. Comparing the aspect ratio distribution of unclosed structures measured from simulations and predicted by the continuum model that accounts for shape fluctuations. The inset shows an example of fitting an ellipse to the vertices on the flattened structure boundary. Simulation parameters: $E_{\mathrm{B}} = 6.0\,k_{\mathrm{B}}T, B = 20\,k_{\mathrm{B}}T, f_{\mathrm{fusion}} = 0.001$, $N = 120$, and the target tubule geometry is (10,0).

the flattened structure to minimize deviations of the vertex positions from the X-Y plane, and fit the positions of the vertices on the structure boundary to an ellipse. The fitting was performed by minimizing a distance function that computes the sum of the distances from vertices on the structure boundary to surface of the trial ellipse. The statistics were collected over 1000 independent simulation trajectories. In the model prediction, the surface energy density σ is obtained from fitting to the $G - N$ free energy curve introduced later in SI section VII, which gives $\sigma \sim 4.5\,k_{\mathrm{B}}T$ per monomer. For the simulation measurements, the mean of the aspect ratio distribution is 1.20, which is close to the aspect ratio of a perfect hexagon (~ 1.15). The standard deviation of the distribution is 0.49. For the model prediction, the mean and standard deviation of the distribution are 1.12 and 0.13 respectively. Comparing these two distributions, we find the spread of the model prediction is narrower and the peak is closer 1 (a perfect circle). We hypothesize this is because the actual pre-closure geometry is a triangulated lattice and it does not have a smooth boundary, contrary to the model assumption.

E. Accounting for shape fluctuations in the discrete model

For the discrete model in the main text, we introduce a cutoff threshold δ to approximately account for shape fluctuations. We use $\delta = 0.05$ for the system with target geometry (10,0). If we assume thet the geometry of the open structure is elliptical, the aspect ratio is 1.1 at the threshold we choose, which is close to the mean of the aspect ratio distribution given by the model prediction. Furthermore, the fraction of structures that fail to close is sensitive to δ. Setting $\delta = 0.05$ leads to a close match between the fraction of structures that fail to close between the discrete model and simulations (see Fig. §7).

F. Comparing the models with and without shape fluctuations to simulation measurements

Figure. §14 compares the tubule diameter distributions estimated from the continuum models with (blue dashed bars) and without (red solid bars) shape fluctuations against simulation measurements ('$\circ$' symbols). We observe that predictions from both of the models accurately estimate the fraction of the distribution near the mean. However, the model which accounts for shape fluctuations more accurately describes the skew measured in simulations, in particular that the skew is biased toward structures that are larger than the mean. We hypothesize that this is because the shape

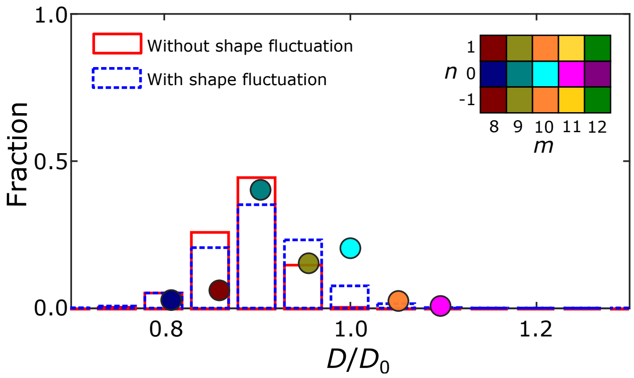

Figure 14. Comparison of tubule width distributions from simulations ('∘' symbols) and the continuum models with (blue dashed bars) and without (red solid bars) and without shape fluctuations. Different face colors represent different assembled tubule geometries. D is the width of the assembled tubule structure. D_0 is the width of the target structure. Simulation parameters: $E_{\mathrm{B}} = 6\,k_{\mathrm{B}}T, B = 20\,k_{\mathrm{B}}T, f_{\mathrm{fusion}} = 0.01, f_{\mathrm{insert}} = 0.0001$, and the target tubule geometry is (10,0). f_{insert} is the attempt frequency of insert/delete move, $f_{\mathrm{insert}} = 0.001$ for all the figures except for this one.

fluctuations allow the pre-closure structures to close to tubule geometries with larger diameters than are accessible to a perfect circle. Therefore, when the size of a pre-closure structure is small, the shape fluctuation would drive the structure to close at a diameter closer to the target diameter D_0 because the closure energy barrier is lower, resulting in a larger mean tubule width when shape fluctuations are accounted for. However, this effect is small and quantitative; the two models give qualitatively similar predictions throughout the parameter space that we have studied.

VI. DEPENDENCE OF THE MEAN TUBULE DIAMETER $\bar{D}$ ON B AND f_{fusion}

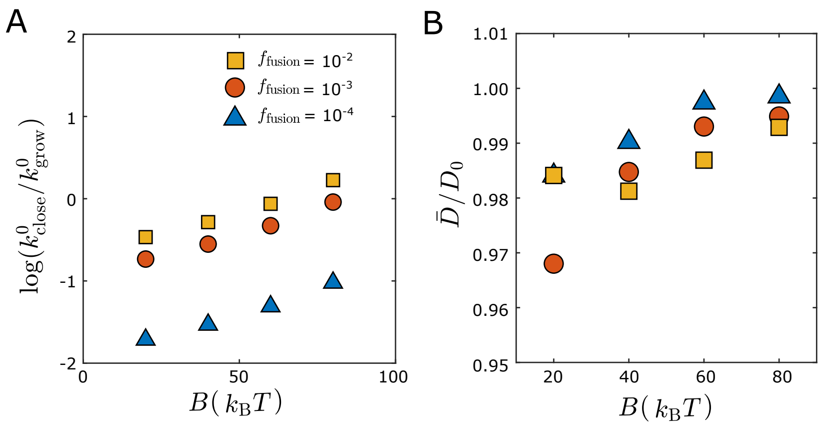

Figure 15. Influence of the bending modulus and edge fusion attempt frequency f_{fusion} on the mean diameter of assembled tubules in simulations. **(A)** The effective closure rate (log scale) as a function of B and f_{fusion} (indicated by symbol type). **(B)** The mean diameter $\bar{D}$ as a function of B. D_0 is the diameter of the ideal (10,0) tubule, the target tubule geometry is (10,0), and $E_{\mathrm{B}} = 6\,k_{\mathrm{B}}T$.

Figure. §15(A) shows that the effective closure rate prefactor (not accounting for the free energy barrier in Eq.(13) of the main text, Eq. (15) of the SI) increases with the bending modulus increases at fixed f_{fusion}. In principle, the faster closure rates favor the tubule width distribution to shift to smaller widths (since the tubule rarely reopens once it closes). However, Figure. §15(B) shows that the mean diameter $\bar{D}$ *increases* as B increases, although the effect is very small. This effect arises because increasing B increases the free energy barrier, thus reducing the closure rate for tubules with diameters that deviate from D_0, thus reducing the probability for premature closure. This elastic energy effect dominates over the prefactor, and shifts the diameter distribution closer to D_0 with increasing B.

VII. THERMODYNAMIC INTEGRATION

We use an adapted thermodynamic integration method to compute the free energies of different tubule geometries. Our primary goal is to estimate the bulk and surface free energy densities that are required for the CNT calculation of the free energy profile as a function of assembly size.

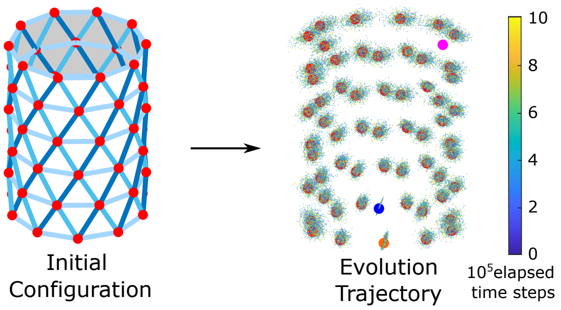

Figure 16. Schematic of the thermodynamic integration implementation. The diagram on the left shows the configuration of the ideal (reference state) cylinder lattice for the Einstein solid. The red circles label the vertices of the ideal lattice for the Einstein solid reference state, while the blue bonds and white faces correspond to the structure of the model. The initial configuration for the simulation has each vertex placed at its ideal cylinder lattice position. The vertex positions then relax via vertex Monte Carlo moves under the Hamiltonian $H(\lambda)$. The diagram on the right illustrates an example trajectory. The large solid circles label the positions of the ideal cylinder lattice vertices, while the clouds of small solid circles label the actual lattice positions at different time steps during the sampling. The color bar of these small solid circles represents the time step. The motions of particles that interact with the magenta, blue, and orange vertices (in the Einstein sold) are constrained to eliminate rigid body motions of the entire structure. The particle interacting with the magenta site is fixed at its ideal lattice position. The particle corresponding to the blue vertex is only allowed to move along the direction that connects between the magenta and the blue vertices. The particle corresponding to the orange vertex is only allowed to move in the plane that contains the magenta, blue, and orange vertices.

A. Thermodynamic integration implementation

We implemented thermodynamic integration as follows. First, we construct an ideal solid lattice corresponding to the helical tubule geometry that our target states are based on. The spatial coordinates of the vertices of the lattice are computed by the method we introduce in the SI section I B. Then, we apply the following Hamiltonian to the system,

$$H(\lambda) = (1-\lambda)U_0 + \lambda U_1 \tag{41}$$

$$U_0 = \frac{1}{2}K \sum_{\text{vertices}} (\vec{r_i} - \vec{r_{0,i}})^2 \tag{42}$$

$$U_1 = \sum_{\text{i}\in\text{Bound Edge Pairs}} -E_{\text{B}} + \frac{1}{2}B(\theta_i - \theta_{0,i})^2 + \sum_{\text{j}\in\text{Edges}} \frac{1}{2}k_{\text{S}}(l_j - l_{0,j})^2 \tag{43}$$

Here λ is a parameter that increases from 0 to 1 during each thermodynamic integration run so that the Hamiltonian changes from U_0 to U_1. U_0 is an Einstein solid potential, in which each particle is connected to the ideal position of the lattice vertex with a spring. $\vec{r_i}$ is the instantaneous position of the ith particle, while $\vec{r_{0,i}}$ is the ideal position of the lattice vertex that the ith particle is connected to. K is the spring constant of the Einstein solid potential. We performed thermodynamic integration computations with $K = 100\,k_{\text{B}}T/l_0^2$. U_1 is the Hamiltonian of our system. In each thermodynamic integration run, we perform Monte Carlo sampling with only vertex moves allowed to equilibrate the system at fixed topology. We perform independent runs with λ ranging from 0 to 0.9 with an increment of 0.05 and from 0.9 to 1.0 with an increment of 0.01. For each λ, we equilibrate the structure by performing 10^6 sweeps, with the number of vertex moves in a sweep equal to the number of vertices N.

The free energy difference between our system and the Einstein solid model is given by

$$F_1 - F_0 = \int_0^1 \langle U_0 - U_1 \rangle_\lambda d\lambda. \tag{44}$$

The integrand is the sample average of the difference between U_0 and U_1 when the system is evolved under the Hamiltonian $H(\lambda)$. U_0 and U_1 are computed by applying the corresponding potential function to the instantaneous spatial configuration in the simulation.

One challenge is that, as $\lambda \to 1$, the Einstein solid potential weakens and the structure undergoes rigid body translations and rotations, which affect U_0 since the coordinates of the ideal lattice are fixed in the lab frame. Therefore, we use the method introduced in the Ref. [9] to prevent rigid body motions of the structure. We constrain the motion of three vertices in the structure as shown in Fig. §16. The particle that interacts with the magenta vertex (in the Einstein solid model) is fixed. The particle that interacts with the blue vertex is only allowed to move along the direction that connects between the magenta and the blue vertices. The particle that interacts with the orange vertex is only allowed to move in the plane that contains the magenta, blue, and orange vertices.

The free energy of the constrained Einstein solid model is given by

$$F_0 = \frac{3}{2}(N_\mathrm{v} - 3)\ln\left(\frac{Kl_0^2}{\pi k_\mathrm{B} T}\right) + \frac{1}{2}\ln\left(\frac{Kl_0^2}{\pi k_\mathrm{B} T}\right) + \ln\left(\frac{Kl_0^2}{\pi k_\mathrm{B} T}\right) = \frac{3}{2}(N_\mathrm{v} - 2)\ln\left(\frac{Kl_0^2}{\pi k_\mathrm{B} T}\right) \tag{45}$$

where N_v is the number of vertices in the structure.

B. Thermodynamic integration results: Free energies of different tubule geometries

We used our thermodynamic integration implementation to compute the free energy ΔG of different tubule geometries assembled with the target geometry (10,0) as a function of the structure size N. Since the number of surface bonds is fixed and finite, the $G-N$ curve should follow a straight line according to CNT. The bulk free energy density at finite temperature $g_{T_\mathrm{finite}}^{(m,n)}$ can then be extracted from the slope of the linear fitting to the $G - N$ curve.

As a comparison against the thermodynamic integration results, we estimate the bulk energy density at zero temperature $g_{T_0}^{(m,n)}$ as the contribution from the elastic energy, given by

$$g_{T_0}^{(m,n)} = -\frac{3}{2}E_\mathrm{B} + \frac{1}{4}B \sum_{i=1,2,3} (\theta_{\mathrm{id},i}^{(m,n)} - \theta_{\mathrm{id},i}^0)^2 \tag{46}$$

in which E_B is the binding energy, i represents different edges of each triangular monomer, $\theta_{\mathrm{id},i}^{(m,n)}$ is the ideal dihedral angle that favours a (m,n) tubule geometry, and $\theta_{\mathrm{id},i}^0$ is the ideal dihedral angle of the target tubule geometry.

The thermodynamic integration results indicate that the free energy difference between different tubule geometries primarily arises due to differences in bending elastic energy. Fig. §17 shows that the free energies $g_{T_\mathrm{finite}}^{(m,n)}$ scale linearly with the elastic energy estimates $g_{T_0}^{(m,n)}$, with slope 1, for all structures that we have considered. As B increases, $g_{T_\mathrm{finite}}^{(m,n)}$ for all tubule geometries generally increases because the entropy loss increases by adding a monomer to the structure. The difference in $g_{T_0}^{(m,n)}$ between different tubule geometries also increases with B because the difference in bending energy increases according to Eq. (46). The inset plots the difference between $g_{T_\mathrm{finite}}^{(m,n)}$ and $g_{T_\mathrm{finite}}^0$ with respect to the diameter D of the tubule, with $B = 20\,k_\mathrm{B}T$. The solid curve is the estimation of the bulk energy density difference Δg^D between a tubule with arbitrary diameter D and the tubule with the same diameter as the (10,0) tubule based on the Helfrich energy model[8]:

$$\Delta g^D = 2Ba_0 \left(\frac{1}{D} - \frac{1}{D_0}\right)^2 \tag{47}$$

in which a_0 is the area of the monomer and D_0 is the diameter of the target tubule. This model only considers the bending energy, yet the free energy density difference $g_{T_\mathrm{finite}}^{(m,n)} - g_{T_\mathrm{finite}}^0$ for most of the discrete tubule geometries falls onto the curved predicted by the model, except for the chiral tubule geometries such as (9,±1) and (10,±1). We hypothesis that this effect reflects the inconsistency between the triangular lattice direction and the curvature direction of the tubule structure itself that arises in chiral structures.

Despite the small geometric effect observed for chiral lattices, the thermodynamic integration results show that the primary factor determining the tubule free energy density is the bending elastic energy difference between a given structure and the target geometry as computed from the continuum Helfrich energy. This result also suggests that the bulk entropy density is similar for different tubule geometries under the same bending rigidity.

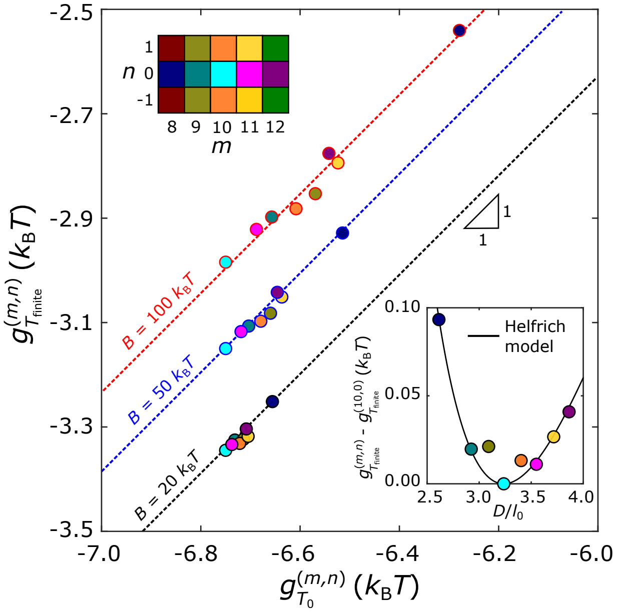

Figure 17. Bulk free energy density of tubule structure (m, n) at finite temperature and zero temperature measured by thermodynamic integration. The target tubule geometry is (10,0) in this computation. Different colors of symbols' face represent different tubule structures (m, n) as shown in the legend. Different colors of symbols' edge represent different bending modulus values B. Dashed lines are guidelines to eyes with slopes of 1. The inset shows the difference between the bulk energy density of an arbitrary tubule geometry (m, n) and the target geometry (10,0) with respect to the diameter D, for $B = 20\,k_\mathrm{B}T$. The solid curve is th continuum prediction based on the Helfrich energy model for the tubule structure with diameter D. In all cases the binding energy $E_\mathrm{B} = 4.5\,k_\mathrm{B}T$.

Control parameters				
	$E_\mathrm{B}/k_\mathrm{B}T$	$B/k_\mathrm{B}T$	Target Structure	f_fusion
Fig.2 Red and blue trajectory	6.0	20	(10,0)	10^{-3}
Fig.2 yellow trajectory	5.0	20	(5,0)	10^{-3}
Fig.3A left/right	6.0	50/100	(10,0)	10^{-3}
Fig.3B left/right	6.0	20	(10,0)/(5,0)	10^{-3}
Fig.3C left/right	6.0	20	(10,0)	$10^{-4}/10^{-2}$
Fig.4 Simulation	6.0	10	(9,4)	10^{-3}
Fig.5 △	6.0	20	(5,0) – (15,0)	10^{-3}
Fig.5 ◯	6.0	40	(5,0) – (15,0)	10^{-3}
Fig.5 □	6.0	60	(5,0) – (15,0)	10^{-3}
Fig.5 ◇	6.0	80	(5,0) – (15,0)	10^{-3}
Fig.6 blue bar	6.0	20	(10,0)	10^{-4}
Fig.6 yellow bar	6.0	20	(10,0)	10^{-2}
Fig.7 △	6.0	20	(10,0)	10^{-4}
Fig.7 ◯	6.0	20	(10,0)	10^{-2}
Fig.7 □	6.0	50	(10,0)	10^{-2}
Fig.7 □	6.0	50	(10,0)	10^{-2}
Fixed parameters				
$k_\mathrm{S}/k_\mathrm{B}T$	$\mu/k_\mathrm{B}T$	f_insert	$f_\mathrm{wedge\ fusion}$	$f_\mathrm{crack\ fusion}$
200	−3	10^{-2}	10^{-4}	10^{-4}

Table I. Simulation parameters in the main text Fig.2 – 7

VIII. SIMULATION PARAMETERS

Table. I presents the parameter values that we used for the simulations shown in the main text. f_{insert}, $f_{\text{wedge fusion}}$ and $f_{\text{crack fusion}}$ are the attempt frequency of the corresponding kinetic moves. (See the following SI Section IX and X for details about the algorithm and the moves).

IX. COMPUTATIONAL MODEL AND KINETIC MONTE CARLO DETAILS

In this section we provide additional details about the model and Monte Carlo simulations that we used to generate the results in the main text. The model and algorithm are closely based on ref. [10] and identical to the one used in ref. [11]. In particular, we consider flexible triangular subunits which can bind to each other along edges with a set of preferred dihedral angles that set the preferred curvatures of the assembling sheet. Monte Carlo simulations are performed in the grand canonical ensemble at fixed μVT, with μ the chemical potential of subunits in the bath. Each Monte Carlo simulation involves a single cluster undergoing assembly and disassembly, with subunits taken from or returned to the bath respectively, as well as structural relaxation moves.

1. *Energies*

In the tubule model, each three edges of the triangular subunits are of different types, $t(p) = 1, 2, 3$, for edge index p and each edge can only bind to an edge of the same type on a neighboring subunit.

The total energy of the system is given by

$$E = \sum_{p}^{3n_{\text{s}}} E^{p}_{\text{stretch}} + \frac{1}{2}\sum_{\langle pq\rangle}\left(E^{pq}_{\text{bend}} + E^{pq}_{\text{bind}}\right) \tag{48}$$

where the first sum goes over all edges, with n_{s} the number of subunits in the cluster. The second sum only goes over bound edges (i.e. non-boundary, adjacent edges, so there are $2n_{\text{b}}$ terms in the sum, with n_{b} as the number of bonds). The 1/2 factor corrects for double counting.

The stretching energy is defined as:

$$E^{p}_{\text{stretch}} = k_{\text{s}}\frac{(l^{p} - l_0)^2}{2} \tag{49}$$

where k_{s} is the stretching modulus, l^p is the instantaneous length, and l_0 is the stress-free (rest) length of an edge. For the tubule model we set the stretching modulus and rest length equal for all edges.

The bending energy is quadratic in deviations from the preferred dihedral angle:

$$E^{pq}_{\text{bend}} = B\frac{\left(\theta^{pq} - \theta_0^{t(p)t(q)}\right)^2}{2} \tag{50}$$

with p and q adjacent edges and $t(p), t(q)$ the edge types. B is the bending modulus and is set equal for all edge types. $\theta_0^{t(p)t(q)}$ is the preferred dihedral angle between edges with types $t(p)$ and $t(q)$. Since only edges of the same types are allowed to bind to each other, $t(p) = t(q) \equiv t$ for all adjacent edge pairs pq, and $\theta_0^{t(p)t(q)} \equiv \theta_0^t$. By adjusting θ_0^t independently for all three edge types $t = 1, 2, 3$ one can tune between different (m, n) target geometries.

The binding energy between two edges p and q (with the same type $t(p) = t(q) = t$) is given by

$$E^{t}_{\text{bind}} = E^{t}_{\text{b}} \tag{51}$$

Binding energies corresponding to all edges are set equal to $E^1_{\text{b}} = E^2_{\text{b}} = E^3_{\text{b}} \equiv E_{\text{b}}$.

In addition to the above terms, each subunit has at its center of mass a spherical excluder of radius $0.2l_0$ to prevent subunit overlaps. Finally, to prevent extreme distortions of subunits, maximum edge length fluctuations are limited to $l_0/2 < l < 3l_0/2$.

2. Coarse-graining

Our model is motivated by the triangular DNA origami subunits developed in Sigl et al.[1], in which subunits bind through lock-and-key 'patches' along subunit edges in which attractive interactions are generated through blunt-end stacking of unsatisfied nucleotides. Therefore, in our model we define attractive bonds along subunit edges. In particular, attractive bonds occur at each shared pair of subunit edges with the same type. Because the interactions in the experimental system are driven by nucleotide stacking, they are extremely short-ranged in comparison to the subunit size (the subunit edge lengths are approximately 60 nm). Therefore, in our simulations we avoid resolving the short length scale fluctuations in separation distance between bound edges and their associated vertices by coarse-graining as follows.

A microstate i is defined as the position of all the $3n_\mathrm{s}$ vertices of n_s subunits: $i \to (\vec{x}_1, \vec{x}_2, ...\vec{x}_{3n_\mathrm{s}})$ The grand canonical probability *density* of finding the system around state i is

$$f(i) = \frac{P(\vec{x}_1, \vec{x}_1 + d\vec{x}_1; ...; \vec{x}_1, \vec{x}_{n_s} + d\vec{x}_{n_s})}{d\vec{x}_1 d\vec{x}_2 ... d\vec{x}_{3n_\mathrm{s}}} = \frac{1}{Z_\Omega} \frac{e^{\beta n_\mathrm{s} \mu}}{\lambda^{9n_\mathrm{s}}} e^{-\beta E_i} \tag{52}$$

where μ is the chemical potential and λ^3 is the standard state volume. This probability density has the dimensions of $1/\mathrm{volume}^{3n_\mathrm{s}}$ corresponding to all the $3n_\mathrm{s}$ vertices of the subunits. Due to bonds, however, some pairs of vertices are confined within a *binding volume* v_a. We consider a square-well potential so that the binding energy is constant within this volume. Analogous to Ref. [10], we can then coarse-grain to avoid resolving intra-bond fluctuations. We assume that fluctuations of bound edges are sufficiently small that each pair of vertices at either end of a bound edge pair are constrained within a *binding volume* v_a. Note that we constrain vertices rather than edges so that the coarse-grained microstate can be represented in terms of positions of vertices rather than edges, which is easier to implement computationally. In the coarse-grained system, a coarse microstate is specified by the coordinates corresponding to the independent vertex degrees of freedom (with 1 degree of freedom for each bound vertex group and unbound vertex): $\Gamma \to (\vec{x}_1, \vec{x}_2, ...\vec{x}_{n_\mathrm{v}})$, where n_v is the number of independent bound vertex groups and free vertices. The probability of such a coarse-grained state is given by the net weight of all the corresponding fine-grained microstates:

$$\rho(\Gamma) = \int_{\{v_\mathrm{a}\}} f(i) \mathrm{d}^{\mathrm{n_{VB}}} \vec{x} \tag{53}$$

where n_VB is the number of vertex-bonds and is given by $n_\mathrm{VB} = 3n_\mathrm{s} - n_\mathrm{v}$. For simplicity, we take the limit in which $\sqrt[3]{v_\mathrm{a}}$ is small in comparison to the length scale over which the elastic energy varies, so that the energy is constant within the bound volume v_a. Then $f(i)$ is a constant, and the probability density is given by

$$\rho(\Gamma) = \frac{1}{Z_\Omega} v_\mathrm{a}^{n_\mathrm{VB}} \frac{e^{\beta n_\mathrm{s} \mu}}{\lambda^{9n_\mathrm{s}}} e^{-\beta E_\Gamma} \tag{54}$$

where E_Γ is the total energy of state Γ (including stretching, bending and binding energies). The coarse graining process is illustrated in Fig.§18.

3. Implementation and data structure

The simulation is implemented on top of the OpenMesh library [12]. Subunits are implemented as triangular mesh elements. OpenMesh uses the halfedge data structure which is suitable to implement triangles with directed normals (Fig.§19). The directed halfedges allow for a clockwise iteration through the boundary of a triangle, which makes the two faces of the triangles distinguishable. Only halfedges with opposite orientations can bind together, making it impossible to form a Mobius strip, for example. The data structure and the resulting iterators in OpenMesh allow for an easy and efficient iteration over the neighborhood of mesh elements (vertices, edges and faces). The implementation of mesh element rearrangements is less straightforward, but we implemented it via the insertion and removal of virtual triangles. In addition, OpenMesh allows for the storage of various properties on mesh elements, allowing storage of edge types and face types stored on the elements. To improve readability in the upcoming sections, we will not represent halfedges separately.

X. THE MONTE CARLO MOVES

In this section we detail the Monte Carlo moves of the simulation. Our algorithm has 11 moves: vertex displacement, simple subunit insertion/deletion, wedge insertion/deletion, wedge fusion/fission, crack fusion/fission, and edge

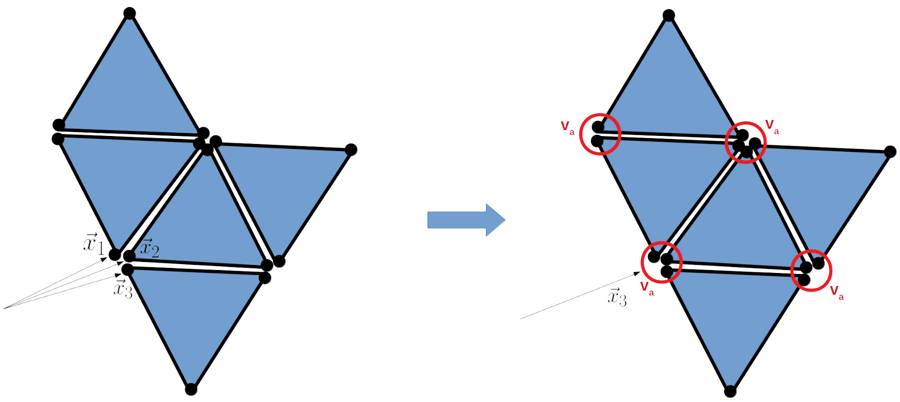

Figure 18. Coarse-graining of an example cluster configuration. In this configuration, the number of subunits is $n_\mathrm{s} = 5$, the number of initial (before coarse-graining) vertices is $3n_\mathrm{s} = 15$, and the number of vertices after coarse-graining is $n_\mathrm{v} = 7$. The red circles indicate bound vertex groups, and the number of vertex degrees of freedom that have been eliminated by coarse-graining in this configuration is $n_\mathrm{VB} = 1 + 3 + 2 + 2 = 8 = 3n_\mathrm{s} - n_\mathrm{v}$. Motivated by DNA origami subunits in Sigl et al. [1], the attractive interactions (i.e. 'bonds') in this model occur along edge-pairs of the same type shared by two subunits. In this configuration there are $n_\mathrm{b} = 4$ bonds.

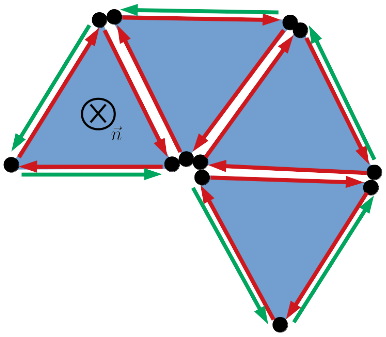

Figure 19. The halfedge data structure used by OpenMesh. Each edge is represented by two directed edges. Boundary edges are no exception and thus are represented by a non-boundary halfedge and a boundary halfedge (in green). This latter is irrelevant for our model. Directed edges allow for the unambiguous definition of face normals, for efficient iterations of the element's neighborhood as well as boundary iterations.

fusion/fission.

Detailed balance. For the transition between state Γ and Γ' detailed balance corresponds to [10, 13]:

$$P(\Gamma) \times \alpha(\Gamma \to \Gamma') \times p_\mathrm{acc}(\Gamma \to \Gamma') = P(\Gamma') \times \alpha(\Gamma' \to \Gamma) \times p_\mathrm{acc}(\Gamma' \to \Gamma) \tag{55}$$

where $\alpha(\Gamma \to \Gamma')$ is the probability of generating a $\Gamma \to \Gamma'$ move attempt (trial), $p_\mathrm{acc}(\Gamma \to \Gamma')$ is the probability of accepting the move, and $P(\Gamma) = \rho(\Gamma)\mathrm{d}^{\mathrm{n_v}(\Gamma)}\vec{x}$ is the equilibrium probability of finding a system in a voxel of volume $\mathrm{d}^{\mathrm{n_v}(\Gamma)}\vec{x}$.

Next, we use Eq. (55) to define the acceptance criteria for each MC move. The acceptance criteria are derived in detail for the wedge fusion/fission move; the steps to follow are the same for all other moves.

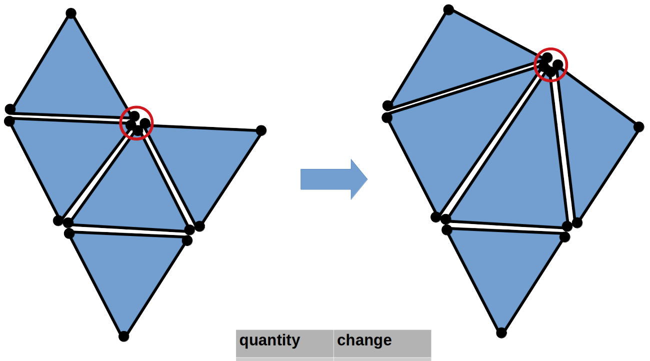

quantity	change
n_s	0
n_{VB}	0
n_b	0

Figure 20. Vertex move. A vertex is randomly displaced and the move is accepted according to the usual Metropolis probability.

A. Vertex displacement

In this move, a vertex is randomly selected, a random uniform displacement is drawn, and the vertex is displaced to its new position according to:

$$x \to x + \mathcal{U}(-d_{\max}, d_{\max}) \tag{56}$$

$$y \to y + \mathcal{U}(-d_{\max}, d_{\max}) \tag{57}$$

$$z \to z + \mathcal{U}(-d_{\max}, d_{\max}) \tag{58}$$

with $d_{\max}$ the maximum displacement. The move is accepted with a probability $p_{\mathrm{acc}} = \exp(-\Delta E/k_{\mathrm{B}}T)$ where ΔE is the (bending plus stretching) energy change due to the displacement. The parameter $d_{\max}$ can be adjusted during a burn-in period to optimize convergence to equilibrium. Generally optimal values are on the order of the typical length scale of thermal fluctuations dictated by the elastic energy, leading to acceptance probabilities on the order of 50%. In our simulations typical values are between $d_{\max} = [0.01l_0, 0.1l_0]$. The vertex displacement move is illustrated in Fig.§20: the number of subunits n_{s}, number of vertices n_{v}, number of vertex bonds n_{VB} and number of bonds n_{b} remains unchanged during this move.

Throughout the article, we define a Monte Carlo *sweep* as the number of steps required for the system to have undergone (on average) n_{v} vertex moves, with n_{v} the number of vertices in the structure. One sweep is one time step in our simulation. The other kinetic moves are attempted less frequently than the vertex moves. Denoting f_{v} as the vertex move attempt frequency, monomer insertion/deletion moves are attempted with frequency $f_{\mathrm{i}} = 10^{-3} f_{\mathrm{v}}$, while wedge fission/fusion and crack fission/fusion moves are attempted with frequency $f_{\mathrm{wf}} = f_{\mathrm{cf}} = 10^{-4} f_{\mathrm{v}}$. Edge fission/fusion moves are attempted as specified in the main text, controlled by the parameter f_{fusion}. The attempt frequency of other moves is fixed throughout all the simulations reported in this work. While the acceptance probabilities are different for different types of moves, they all guarantee detailed balance as shown below.

B. Simple insertion / removal

1. *Simple insertion*

In this move, an edge is randomly selected from the set of all boundary edges, where a new subunit will be attached. The number of such boundary edges is n_{e}. Subunits can be inserted in n_{r} different rotations, where n_{r} is the number of distinct rotational states for a subunit which has one edge aligned with the edge of a neighboring subunit. For our triangular subunits with three distinct edge types, $n_{\mathrm{r}} = 3$. In our algorithm, during insertion of a subunit its rotational state is chosen randomly from the set of three possibilities. If the aligned edge is not complementary to the type of the boundary edge, then the move is rejected. In this work, the two edges must be of the same type to be complementary.

The positions of two of the new subunit's vertices (those at either end of the edge being bound) are set equal to the positions of the corresponding vertices of the boundary edge to which it is binding. The third vertex position is randomly chosen from within a volume v_{add} centered at the equilibrium position of the new vertex.

Thus, the attempt probability for a simple insertion is given by:

$$\alpha(i \to j) = n_{\text{e}} f_{\text{i}} \tau n_{\text{r}} \times \frac{1}{n_{\text{e}} n_{\text{r}} (v_{\text{add}}/\text{d}\vec{x})}. \tag{59}$$

Then, applying Eq. (55) and the attempt probability for the reverse move (simple deletion, presented next, Eq. (61)), the acceptance probabilities for a simple insertion is

$$p_{\text{acc}}(i \to j) = \min\left[1, \frac{v_{\text{a}}^2 v_{\text{add}}}{\lambda^9} \exp[-(\Delta E_{i\to j} - \mu)/k_{\text{B}}T]\right]. \tag{60}$$

$\Delta E_{i\to j}$ is the energy change due to the move and includes the stretching energy of the newly inserted subunit, its bending energy along the shared edge, and the binding energy due to the creation of an extra bond. During this move, one new (edge) bond and two new vertex bonds are created; i.e. $n_{\text{b}} \to n_{\text{b}} + 1$ and $n_{\text{VB}} \to n_{\text{VB}} + 2$. Moreover, the number of vertices in the structure increases by one, $n_{\text{v}} \to n_{\text{v}} + 1$.

2. Simple removal

The reverse move to simple insertion is simple removal. Subunits that can be deleted with this move are those with two boundary edges. The number of simply removable subunits is n_{sr}. One of these is selected randomly, so the attempt probability is

$$\alpha(j \to i) = n_{\text{sr}} f_{\text{i}} \tau \times \frac{1}{n_{\text{sr}}} \tag{61}$$

and, using Eq. (55) and Eq. (59), the acceptance probability is

$$p_{\text{acc}}(j \to i) = \min\left[1, \frac{\lambda^9}{v_{\text{a}}^2 v_{\text{add}}} \exp[-(\Delta E_{j\to i} + \mu)/k_{\text{B}}T]\right] \tag{62}$$

During this move, the structure loses one (edge) bond and two vertex bonds; $n_{\text{b}} \to n_{\text{b}} - 1$ and $n_{\text{VB}} \to n_{\text{VB}} - 2$. The number of vertices in the structure decreases by one, $n_{\text{v}} \to n_{\text{v}} - 1$.

If there are multiple species with chemical potentials μ_k, detailed balance must be satisfied for each species, individually. Moreover, each species can have different insertion rates f_{i}^k.

To keep $\alpha < 1$, we ensure that the insertion rate f_{i} constrained by

$$n_{\text{e}} f_{\text{i}} \tau n_{\text{r}} < 1 \tag{63}$$

$$n_{\text{sr}} f_{\text{i}} \tau < 1 \tag{64}$$

In equilibrium, one can use adaptive rates, i.e. reduce k_i on the run if the above condition is not satisfied. In that case, sampling is not taken for the ensuing several time steps. Alternatively, the rates may be set to a low enough value from the beginning and only tested on the run to ensure that the $\alpha < 1$ condition is satisfied. This latter technique is appropriate for dynamical runs as it keeps the rates constant throughout the simulation.

Moreover, we must ensure that v_{add} is large enough so that the vertex does not leave the v_{add} volume during structural relaxation moves; otherwise the insertion/deletion moves would not be reversible and the detailed balance would be violated. For a better convergence, one could choose a gaussian distribution $\mathcal{N}(\vec{r})$ for the position of the new vertex instead of a uniform distribution $1/v_{\text{add}}$. In this case, this distribution has to be accounted for in the acceptance probabilities $p_{\text{acc}}(i \to j)$ and $p_{\text{acc}}(j \to i)$.

C. Wedge insertion/removal

1. Wedge insertion

Wedges are positions in the structure where a triangle can be inserted via attaching to two edges (Fig. §22). In a wedge move, we pick randomly from the set of available wedge positions in the structure, and pick a random

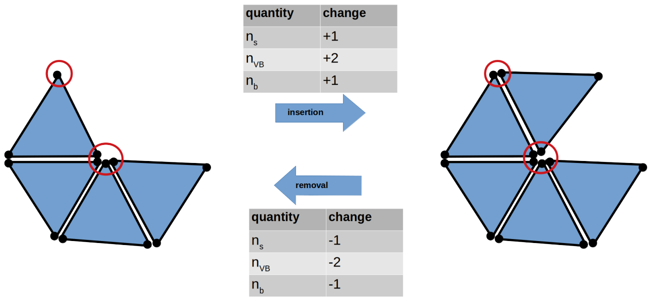

Figure 21. Simple insertion and removal.

orientation for the new subunit. Denoting the number of wedge positions in a given structure as n_{w}, the attempt probability for a wedge move is

$$\alpha(i \to j) = n_{\mathrm{w}} f_{\mathrm{i}} \tau n_{\mathrm{r}} \times \frac{1}{n_{\mathrm{r}} n_{\mathrm{w}}} \tag{65}$$

In contrast to the simple insertion move, there is no need for random vertex displacement in a wedge move because all three vertices of the new subunit are fixed by the three vertices of the wedge position. Combining Eq. (65) and the attempt probability for wedge removal (Eq. (67)), The acceptance probability for a wedge insertion is

$$p_{\mathrm{acc}}(i \to j) = \min\left[1, \frac{v_{\mathrm{a}}^3}{\lambda^9} \exp[-(\Delta E_{i\to j} - \mu)/k_{\mathrm{B}}T]\right]. \tag{66}$$

During a wedge insertion, two edge bonds and three vertex bonds are created; i.e., $n_{\mathrm{b}} \to n_{\mathrm{b}} + 2$ and $n_{\mathrm{VB}} \to n_{\mathrm{VB}} + 3$, but the number of vertices is unchanged, $n_{\mathrm{v}} \to n_{\mathrm{v}}$. $\Delta E_{i\to j}$ includes the binding energy of the two newly formed bonds, the two bending energies along the two newly bound edges and the stretching energy of the newly inserted subunit.

2. Wedge removal

The reverse move of wedge insertion is wedge removal. In a wedge removal, we randomly choose one of the removable wedges from a given structure. With the number of removable wedges as n_{wr}, the attempt probability is

$$\alpha(j \to i) = n_{\mathrm{wr}} f_{\mathrm{i}} \tau \times \frac{1}{n_{\mathrm{wr}}}. \tag{67}$$

Using Eq. (65), the acceptance probability for a wedge removal is then

$$p_{\mathrm{acc}}(j \to i) = \min\left[1, \frac{\lambda^9}{v_{\mathrm{a}}^3} \exp[-(\Delta E_{j\to i} + \mu)/k_{\mathrm{B}}T]\right]. \tag{68}$$

We have the following constraints on rates f_{i} for wedge insertion/removal:

$$n_{\mathrm{w}} f_{\mathrm{i}} \tau n_{\mathrm{r}} < 1 \tag{69}$$

$$n_{\mathrm{wr}} f_{\mathrm{i}} \tau < 1 \tag{70}$$

As for simple insertion and removal, in the case of multiple species, detailed balance is satisfied for each species separately for wedge insertion/removal.

D. Wedge fusion / fission

1. Wedge fusion

In this move, a *fusable wedge* is closed, without inserting a new subunit (Fig.§23). That is, the two vertices on either side of the wedge opening are merged into a single vertex. Fusable wedges are vertex pairs that i) form a wedge (as in the case of wedge insertion) and ii) are within a separation distance of l_{fuse}.

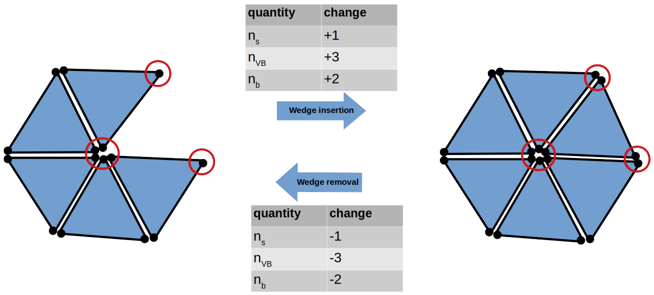

Figure 22. Wedge insertion and removal.

Denoting the number of fusible wedge positions as n_{w}, in each MC step, a wedge fusion is attempted with probability $n_{\mathrm{w}} f_{\mathrm{wf}} \tau$, where f_{wf} is an adjustable parameter controlling the relative probability of attempting wedge fusion. Then, a wedge position is selected randomly from the set of all n_{w} fusible wedges. The attempt probability is thus

$$\alpha(i \to j) = n_{\mathrm{w}} f_{\mathrm{wf}} \tau \times \frac{1}{n_{\mathrm{w}}}. \tag{71}$$

Using Eqs. (71) and (73), the acceptance probability for fusion moves is

$$p_{\mathrm{acc}}(i \to j) = \min\left[1, \frac{v_{\mathrm{a}}}{v_{\mathrm{fuse}}} \exp(-\Delta E_{i\to j}/k_{\mathrm{B}}T)\right] \tag{72}$$

where $v_{\mathrm{fuse}} = (4\pi/3)(l_{\mathrm{fuse}}/2)^3$ is the volume of a sphere with diameter l_{fuse}, and $\Delta E_{i\to j}$ is the energy change due to the fusion, including changes in bending, stretching, and binding energies. A fusion move increases the number of edge bonds and vertex bonds by one, $n_{\mathrm{b}} \to n_{\mathrm{b}}+1$ and $n_{\mathrm{VB}} \to n_{\mathrm{VB}}+1$; the factor of v_{a} appears in Eq. (72) to account for the latter.

2. *Wedge fission*

Wedge fission, in which a wedge is opened, is the reverse of the wedge fusion move. Fissionable edges are those edges that can be split along their boundary vertex to obtain a wedge. Denoting the number of such edges as n_{f}, the probability of attempting a wedge fission move during an MC step is $n_{\mathrm{f}} f_{\mathrm{wf}} \tau$. If a fission move is attempted, then an edge is selected randomly from the n_{f} fissionable edges. The position of one of the new vertices is selected randomly within the sphere of volume v_{fuse} centered at the original position of the merged vertices, and the other new vertex is placed in the opposite direction from the original position, at the same distance. Thus, the attempt generation probability is

$$\alpha(j \to i) = n_{\mathrm{f}} f_{\mathrm{wf}} \tau \times \frac{1}{n_{\mathrm{f}}(v_{\mathrm{fuse}}/\mathrm{d}\vec{x})} \tag{73}$$

and the acceptance probability is

$$p_{\mathrm{acc}}(j \to i) = \min\left[1, \frac{v_{\mathrm{fuse}}}{v_{\mathrm{a}}} \exp(-\Delta E_{j\to i}/k_{\mathrm{B}}T)\right] \tag{74}$$

We verify that detailed balance holds between wedge fusion and fission as follows. There are two cases to consider:

1. $(v_{\mathrm{fuse}}/v_{\mathrm{a}}) \exp(-\Delta E_{j\to i}/k_{\mathrm{B}}T) < 1 \Leftrightarrow (v_{\mathrm{a}}/v_{\mathrm{fuse}}) \exp(-\Delta E_{i\to j}/k_{\mathrm{B}}T) > 1$

   In this case, $p_{\mathrm{acc}}(i \to j) = 1$ and $p_{\mathrm{acc}}(j \to i) = (v_{\mathrm{fuse}}/v_{\mathrm{a}}) \exp(-\Delta E_{j\to i}/k_{\mathrm{B}}T)$. Then

$$P_i \times \alpha(i \to j) \times p_{\mathrm{acc}}(i \to j) = \frac{1}{Z_\Omega} v_{\mathrm{a}}^{n_{\mathrm{VB},i}} \exp[-(E_i - \mu n_{s,i})/k_{\mathrm{B}}T] \frac{1}{\lambda^{9n_{s,i}}} \times \mathrm{d}^{n_{\mathrm{v},i}}\vec{x} \times k_{\mathrm{wf}}\tau \tag{75}$$

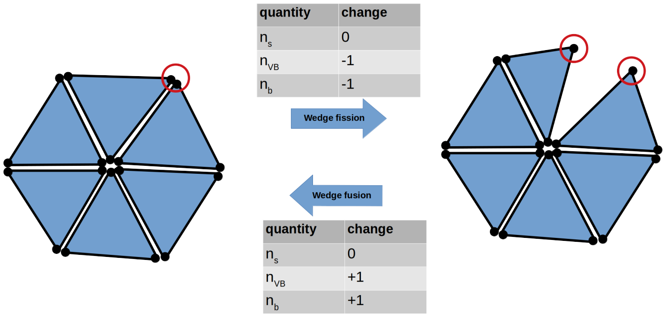

Figure 23. Wedge fusion and fission.

and

$$P_j \times \alpha(j \to i) \times p_{\rm acc}(j \to i) = \frac{1}{Z_\Omega} v_{\rm a}^{n_{{\rm VB},j}} \exp[-(E_j - \mu n_{s,j})/k_{\rm B}T] \frac{1}{\lambda^{9n_{s,j}}} \times {\rm d}^{\rm n_{v,j}}\vec{x} \tag{76}$$

$$\times\, k_{\rm wf}\tau {\rm d}\vec{x}/v_{\rm fuse} \times (v_{\rm fuse}/v_{\rm a}) \exp(-\Delta E_{j\to i}/k_{\rm B}T) \tag{77}$$

Using: $\Delta E_{j\to i} = E_i - E_j$, $n_{s,i} = n_{s,j}$ (because the move leaves the subunit number unchanged), $n_{VB,i} = n_{{\rm VB},j} - 1$ (one vertex bond is broken upon fission) and $n_{v,i} = n_{v,j} + 1$ (an extra vertex is being born upon fission), we see that the two are equal and detailed balance holds.

2. $(v_{\rm fuse}/v_{\rm a}) \exp(-\Delta E_{j\to i}/k_{\rm B}T) > 1 \Leftrightarrow (v_{\rm a}/v_{\rm fuse}) \exp(-\Delta E_{i\to j}/k_{\rm B}T) < 1$

   In this case, $p_{\rm acc}(i \to j) = (v_{\rm a}/v_{\rm fuse}) \exp(-\Delta E_{i\to j}/k_{\rm B}T)$ and $p_{\rm acc}(j \to i) = 1$. Then

$$P_i \times \alpha(i \to j) \times p_{\rm acc}(i \to j) = \frac{1}{Z_\Omega} v_a^{n_{{\rm VB},i}} \exp[-(E_i - \mu n_{s,i})/k_{\rm B}T] \frac{1}{\lambda^{9n_{s,i}}} \times {\rm d}^{\rm n_{v,i}}\vec{x} \times k_{\rm wf}\tau \tag{78}$$

$$\times\, (v_{\rm a}/v_{\rm fuse}) \exp(-\Delta E_{i\to j}/k_{\rm B}T) \tag{79}$$

and

$$P_j \times \alpha(j \to i) \times p_{\rm acc}(j \to i) = \frac{1}{Z_\Omega} v_a^{n_{{\rm VB},j}} \exp[-(E_j - \mu n_{s,j})/k_{\rm B}T] \frac{1}{\lambda^{9n_{s,j}}} \times {\rm d}^{\rm n_{v,j}}\vec{x} \tag{80}$$

$$\times\, k_{\rm wf}\tau {\rm d}\vec{x}/v_{\rm fuse} \tag{81}$$

Using again $\Delta E_{j\to i} = E_i - E_j$, $n_{s,i} = n_{s,j}$, $n_{VB,i} = n_{{\rm VB},j} - 1$ and $n_{v,i} = n_{v,j} + 1$, detailed balance holds.

Note that detailed balance is satisfied regardless of the values of $f_{\rm wf}\tau$ or $v_{\rm fuse}$, but as with all of the move frequencies these parameters can be optimized during burn-in to accelerate convergence to the equilibrium distribution $P(i)$. In our simulations, we find that the optimal value of $v_{\rm fuse}$ is on the order of the optimal value of $d_{\rm max}$ for analogous reasons: if $v_{\rm fuse}$ is too small there will be very few vertex pairs identified as fusable, so $n_{\rm w}$ will be low. If $v_{\rm fuse}$ is too large, there will be many fusion candidates but most fusion attempts will be rejected due to the large elastic energy change necessary for the merging deformation.

Most importantly, we note the constraint on the parameters $f_{\rm wf}\tau$ to ensure that generation probabilities do not become larger than unity. Because each attempt is generated as a three step process, using three probabilities, one has to ensure that all those probabilities are less than 1. Specifically,

$$n_{\rm w} f_{\rm wf}\tau < 1 \tag{82}$$

$$n_{\rm f} f_{\rm wf}\tau < 1. \tag{83}$$

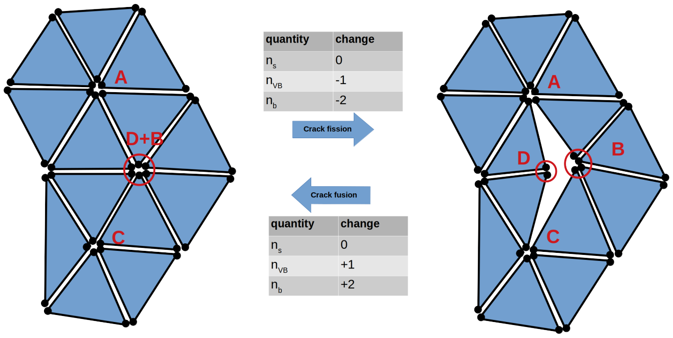

Figure 24. Crack fusion and fission.

E. Crack fusion / fission

1. *Crack fusion*

Crack fusion closes a crack within the structure; i.e., two adjacent pairs of edges are merged (Fig.§24). Cracks are identified as 4-edge-length holes inside the structure. If the vertices of the hole are labeled A, B, C, D then the polygon ABCD forms a closed loop (see Fig.§24). The crack can be closed by either merging vertices A and C (and correspondingly edges CD to DA and AB to BC) or by merging vertices B and D (and correspondingly edges AD to AB and CD to CB). Each 4-edge-length loop thus defines two potential fusable cracks. However, an additional condition for a crack to be fusable is that its merging vertices must be within a distance l_{fuse} (A and C or D and B in this example). In this work, we have set the crack fusion volume to be the same as that for wedge fusion to reduce the number of parameters, but it is not necessary that they be the same.and the acceptance probability is

$$p_{\mathrm{acc}}(i \to j) = \min\left[1, \frac{v_{\mathrm{a}}}{v_{\mathrm{fuse}}}\exp(-\Delta E_{i\to j}/k_{\mathrm{B}}T)\right] \tag{84}$$

There are two edge bonds and one vertex bond formed during a crack fusion.

2. *Crack fission*

The reverse move for crack fusion is crack fission. With the number of potential cracks as n_{cf}:

$$\alpha(j \to i) = n_{\mathrm{cf}} f_{\mathrm{cf}} \tau \times \frac{1}{n_{\mathrm{cf}}(v_{\mathrm{fuse}}/\mathrm{d}\vec{x})} \tag{85}$$

$$p_{\mathrm{acc}}(j \to i) = \min\left[1, \frac{v_{\mathrm{fuse}}}{v_{\mathrm{a}}}\exp(-\Delta E_{j\to i}/k_{\mathrm{B}}T)\right] \tag{86}$$

As for the case of wedge fusion/fission, the crack fusion attempt frequency parameter f_{cf} is constrained by the conditions maintaining probabilities smaller than unity:

$$n_{\mathrm{c}} f_{\mathrm{cf}} \tau < 1 \tag{87}$$

$$n_{\mathrm{cf}} f_{\mathrm{cf}} \tau < 1 \tag{88}$$

(89)

F. Edge fusion / fission

1. *Edge fusion*

During this move two non-neighbor edges are fused (Fig.§25). Fusable edges are non-neighboring edge pairs whose corresponding vertices are within a separation distance l_{fuse}. Since edges are directed, they can only fuse such that,

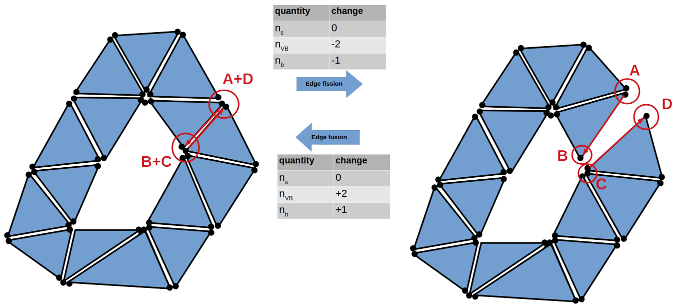

Figure 25. Edge fusion and fission.

after fusion, they point in the opposite direction. Assuming the edges to be fused are $A \to B$ and $C \to D$ (see Fig.§25), vertex A will merge into vertex D and vertex B will merge into vertex C. Edges are counted as fusable if A is within a distance $l_{\rm fuse}$ to D and B is also within a distance $l_{\rm fuse}$ to C. The attempt probability is analogous to that for wedge and crack fusion/fission,

$$\alpha(i \to j) = n_{\rm e} f_{\rm fusion} \tau \times \frac{1}{n_{\rm e}} \tag{90}$$

with $n_{\rm e}$ the number of fusable edges and $f_{\rm fusion}$ the edge fusion frequency parameter. The acceptance probability is

$$p_{\rm acc}(i \to j) = \min \left[1, \left(\frac{v_{\rm a}}{v_{\rm fuse}} \right)^2 \exp(-\Delta E_{i \to j}/k_{\rm B}T) \right] \tag{91}$$

During edge fusion, one edge bond and two vertex bonds are created.

2. Edge fission

Edge fission is the reverse move of edge fusion. $n_{\rm ef}$ is the number of breakable edges, that is, those edges that have both vertices on the boundary and which would not result in breaking the structure apart.

$$\alpha(j \to i) = n_{\rm ef} f_{\rm fusion} \tau \times \frac{1}{n_{\rm ef}(v_{\rm fuse}/{\rm d}\vec{x})^2} \tag{92}$$

The factor $1/(v_{\rm fuse})^2$ arises because we must select a random position for each pair of vertices, independently. The acceptance probability is then

$$p_{\rm acc}(j \to i) = \min \left[1, \left(\frac{v_{\rm fuse}}{v_{\rm a}} \right)^2 \exp(-\Delta E_{j \to i}/k_{\rm B}T) \right]. \tag{93}$$

To maintain probabilities within unity, the edge fusion frequency parameter $f_{\rm fusion}$ is constrained by

$$n_{\rm e} f_{\rm fusion} \tau < 1 \tag{94}$$
$$n_{\rm ef} f_{\rm fusion} \tau < 1. \tag{95}$$

XI. SUPPLEMENTARY VIDEOS

Movie S1. Kinetic Monte Carlo simulation trajectory corresponding to the red trajectory in Fig.1: $E_{\rm B} = 6.0\ k_{\rm B}T, B = 20\ k_{\rm B}T, f_{\rm fusion} = 0.01$, target structure is (10,0).
Movie S2. Kinetic Monte Carlo simulation trajectory corresponding to the blue trajectory in Fig.1: $E_{\rm B} = 6.0\ k_{\rm B}T, B = 20\ k_{\rm B}T, f_{\rm fusion} = 0.01$, target structure is (10,0).

Movie S3. Kinetic Monte Carlo simulation trajectory corresponding to the yellow trajectory in Fig.1: $E_{\mathrm{B}} = 5.0\ k_{\mathrm{B}}T, B = 20\ k_{\mathrm{B}}T, f_{\mathrm{fusion}} = 0.01$, target structure is (5,0). The playback speed is 10 times faster than Movie S1 and Movie S2.

[1] Christian Sigl, Elena M. Willner, Wouter Engelen, Jessica A. Kretzmann, Ken Sachenbacher, Anna Liedl, Fenna Kolbe, Florian Wilsch, S. Ali Aghvami, Ulrike Protzer, Michael F. Hagan, Seth Fraden, and Hendrik Dietz. Programmable icosahedral shell system for virus trapping. *Nature Materials*, 20(9):1281–1289, September 2021. Bandiera_abtest: a Cg_type: Nature Research Journals Number: 9 Primary_atype: Research Publisher: Nature Publishing Group Subject_term: Nanobiotechnology;Nanoscale biophysics;Nucleic-acid therapeutics;Self-assembly;Synthetic biology Subject_term_id: nanobiotechnology;nanoscale-biophysics;nucleic-acid-therapeutics;self-assembly;synthetic-biology.

[2] Daichi Hayakawa, Thomas E. Videbæk, Douglas M. Hall, Huang Fang, Christian Sigl, Elija Feigl, Hendrik Dietz, Seth Fraden, Michael F. Hagan, Gregory M. Grason, and W. Benjamin Rogers. Geometrically programmed self-limited assembly of tubules using DNA origami colloids. *arXiv:2203.01421 [cond-mat]*, March 2022. arXiv: 2203.01421.

[3] Richard K. F. Lee, Barry J. Cox, and James M. Hill. An exact polyhedral model for boron nanotubes. *Journal of Physics A: Mathematical and Theoretical*, 42(6):065204, 2009. Publisher: IOP Publishing.

[4] H. S. Seung and David R. Nelson. Defects in flexible membranes with crystalline order. *Physical Review A*, 38(2):1005–1018, July 1988. Publisher: American Physical Society.

[5] Michael F. Hagan and Gregory M. Grason. Equilibrium mechanisms of self-limiting assembly. *Reviews of Modern Physics*, 93(2):025008, June 2021. Publisher: American Physical Society.

[6] Michael F. Hagan, Oren M. Elrad, and Robert L. Jack. Mechanisms of kinetic trapping in self-assembly and phase transformation. *Journal of Chemical Physics*, 135(10), September 2011.

[7] S. Whitelam and R. L. Jack. The Statistical Mechanics of Dynamic Pathways to Self-assembly. *Ann Rev Phys Chem*, 66:143–63, 2015.

[8] Helfrich, W. Size distributions of vesicles : the role of the effective rigidity of membranes. *J. Phys. France*, 47(2):321–329, 1986.

[9] Evan Pretti, Hasan Zerze, Minseok Song, Yajun Ding, Runfang Mao, and Jeetain Mittal. Size-dependent thermodynamic structural selection in colloidal crystallization. *Science Advances*, 5(9):eaaw5912, September 2019.

[10] Grant M. Rotskoff and Phillip L. Geissler. Robust nonequilibrium pathways to microcompartment assembly. *Proceedings of the National Academy of Sciences of the United States of America*, 115(25):6341–6346, 2018.

[11] Botond Tyukodi, Farzaneh Mohajerani, Douglas M. Hall, Gregory M. Grason, and Michael F. Hagan. Thermodynamic size control in curvature-frustrated tubules: Self-limitation with open boundaries. *arXiv:2109.01174 [cond-mat]*, September 2021. arXiv: 2109.01174.

[12] M Botsch, S Steinberg, S Bischoff, and L Kobbelt. OpenMesh – a generic and efficient polygon mesh data structure. In *OpenSG Symposium*, 2002.

[13] Daan Frenkel and Berend Smit. *Understanding Molecular Simulation: From Algorithms to Applications*. Elsevier, October 2001.
